# Supplementary material for: Antimicrobial protein REG3A regulates glucose homeostasis and insulin resistance in obese diabetic mice
Source: Commun Biol. 2023 Mar 15;6:269. doi: 10.1038/s42003-023-04616-5 (PMC10015038; doi:10.1038/s42003-023-04616-5)

Supplementary Figure 1

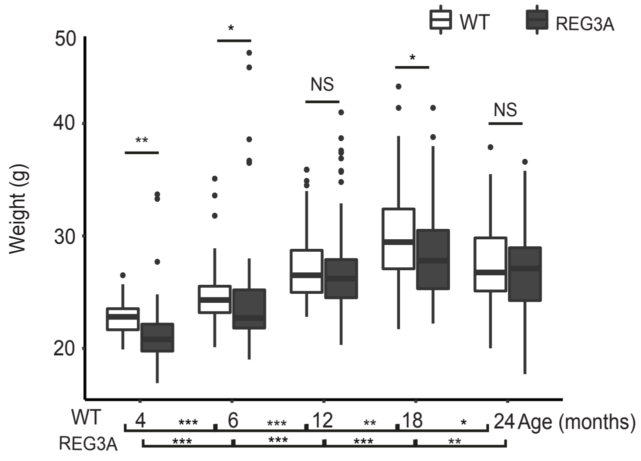

Supplementary Figure 2

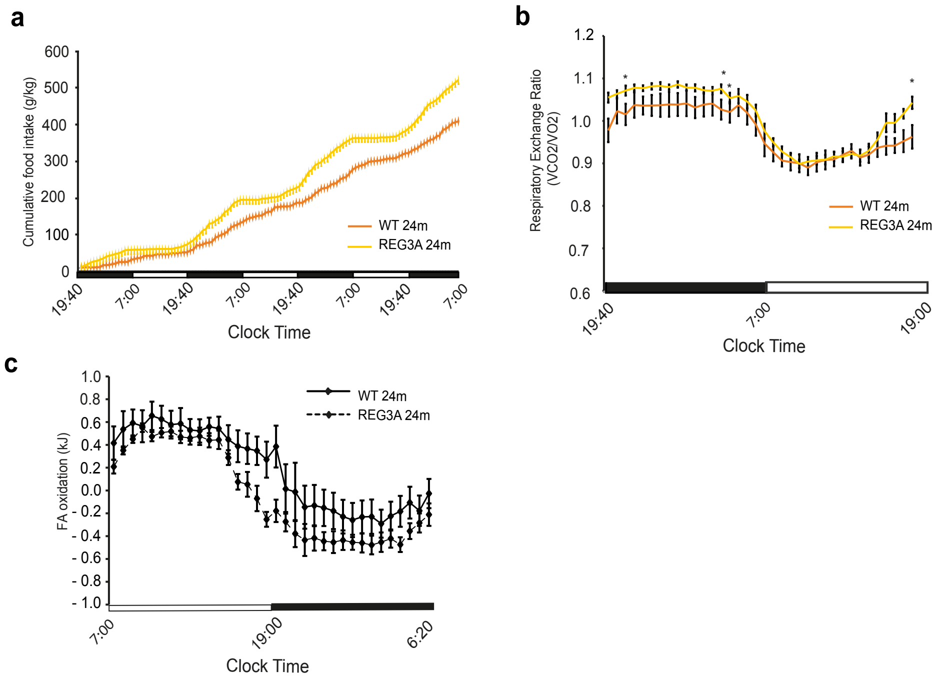

Supplementary Figure 3

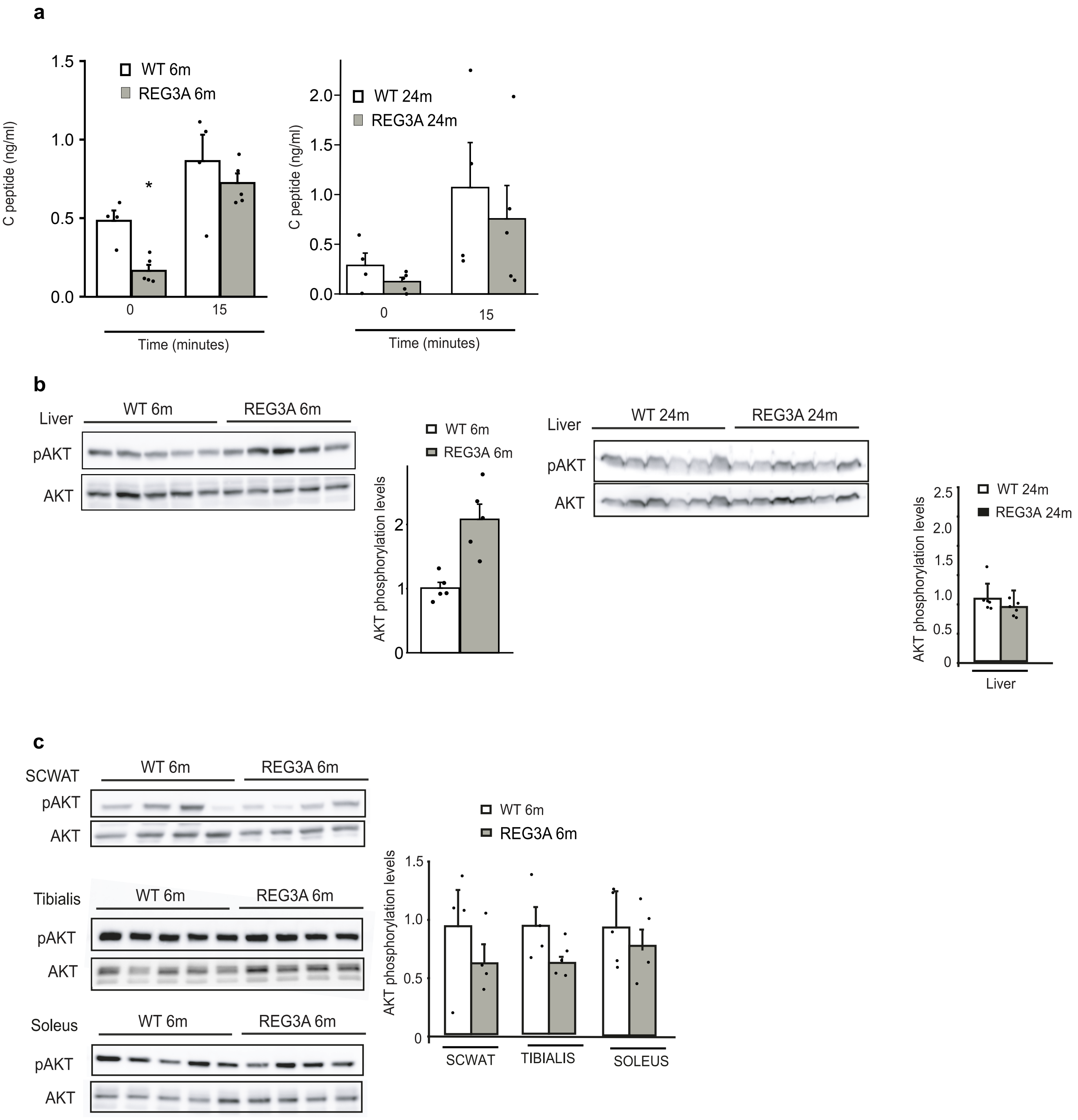

Supplementary Figure 4

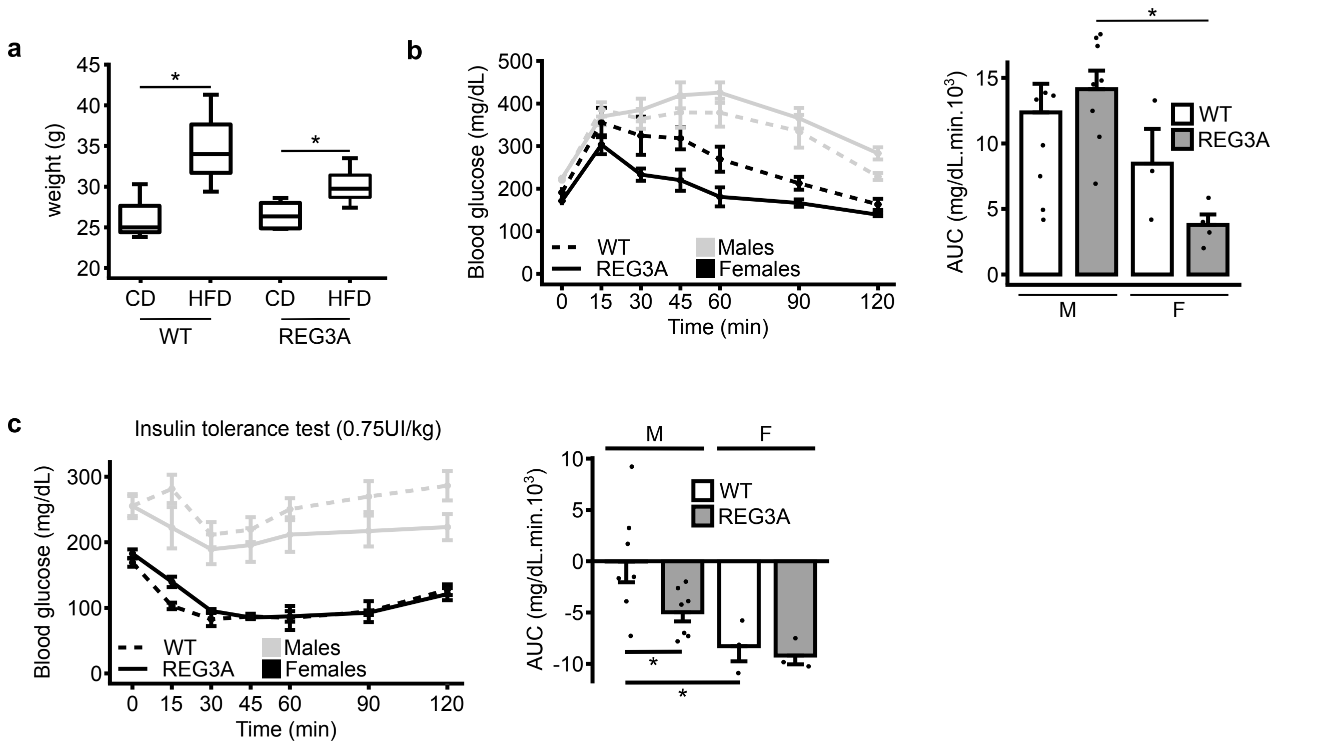

Supplementary Figure 5

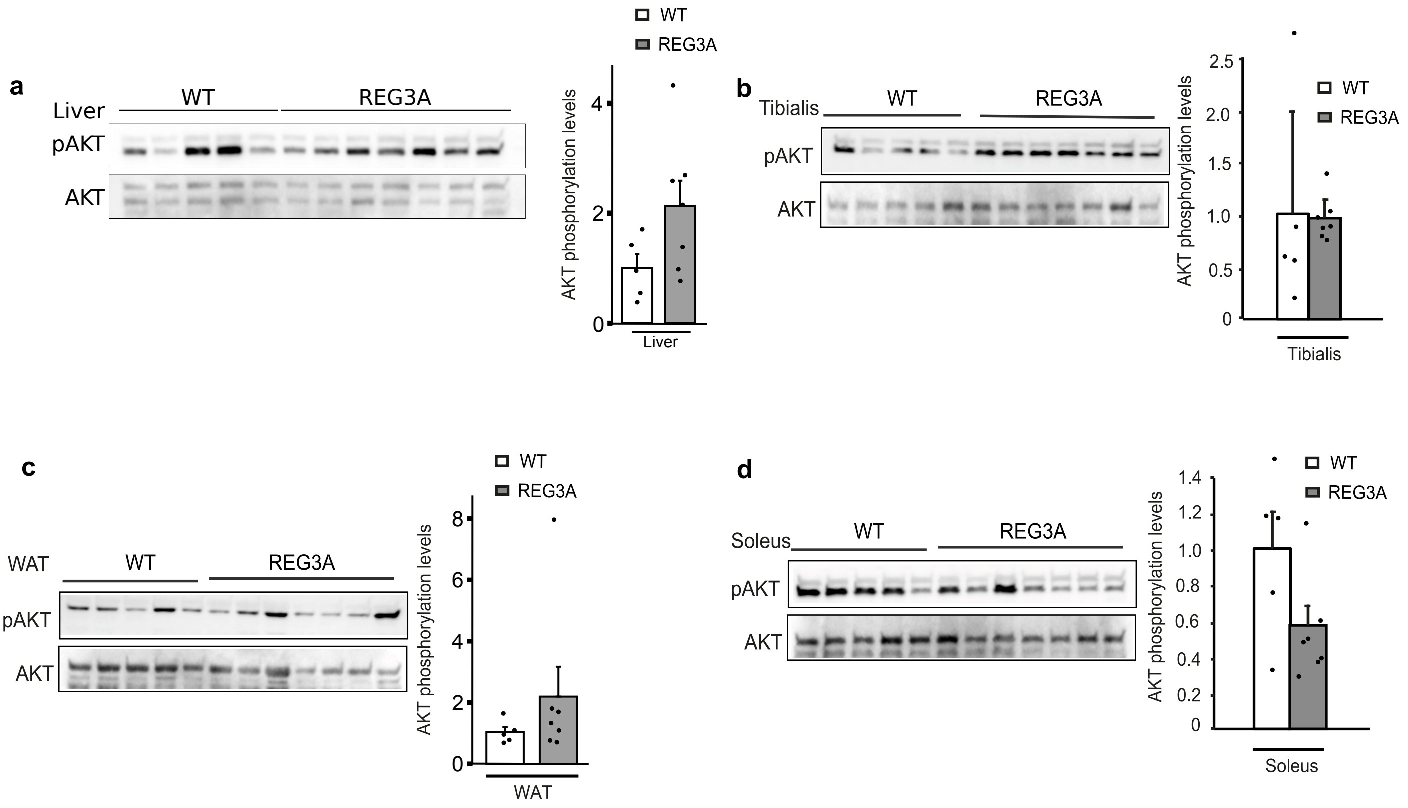

Supplementary Figure 6

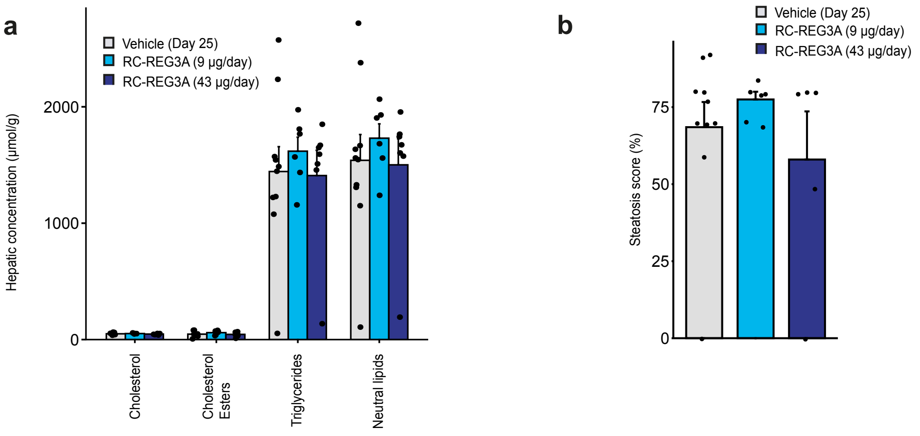

Supplementary Figure 7

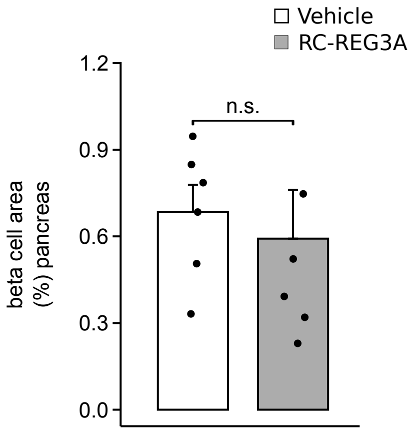

Supplementary Fig. 7. REG3A does not alter pancreatic islets under a high fat diet. Quantification of insulin-producing islet surface area in WT mice fed a high-fat diet that received 43μg per day of a recombinant REG3A protein (rcREG3A) subcutaneously or an equivalent volume of buffer (vehicle) for 28 days. The data are averages ± SEM. NS, no significance (Student's t-test).

Supplementary Figure 8

a

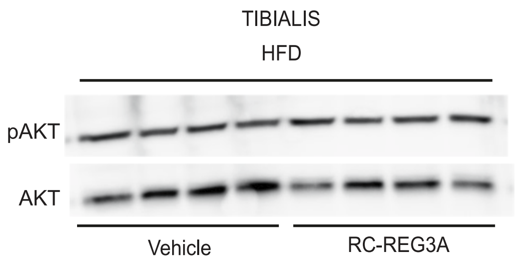

b

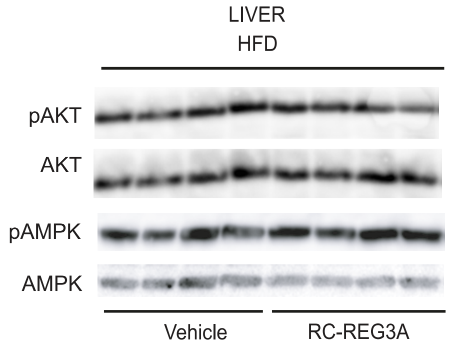

Supplementary Figure 9

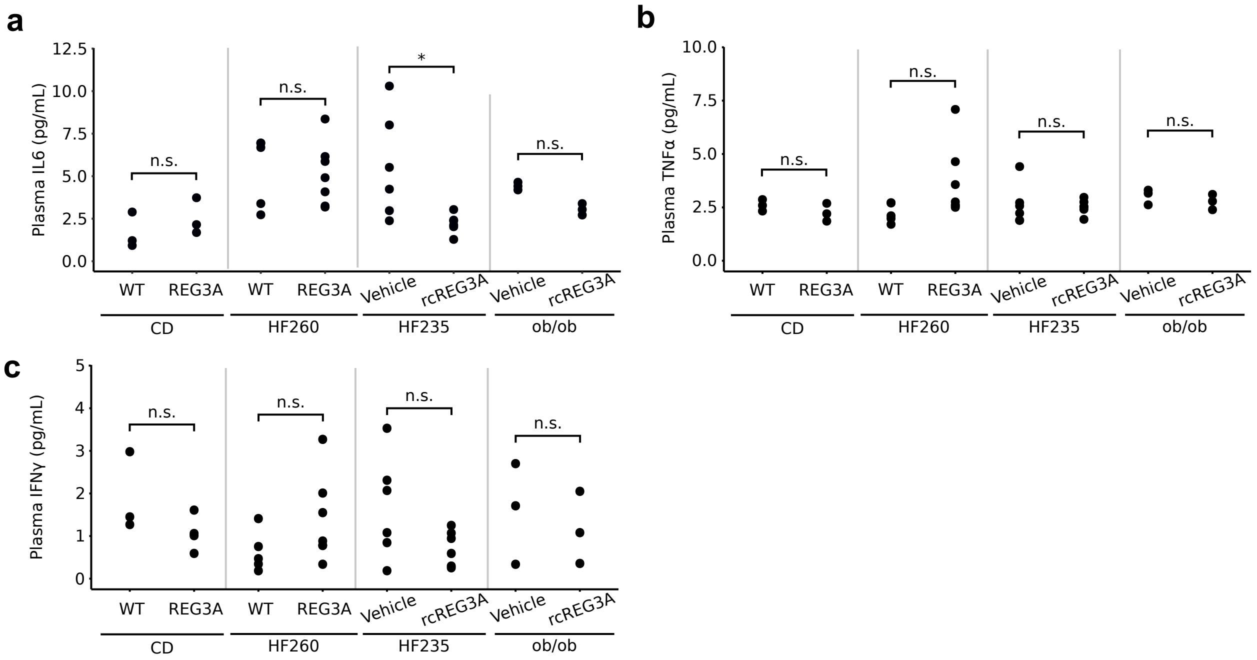

Supplementary Figure 10

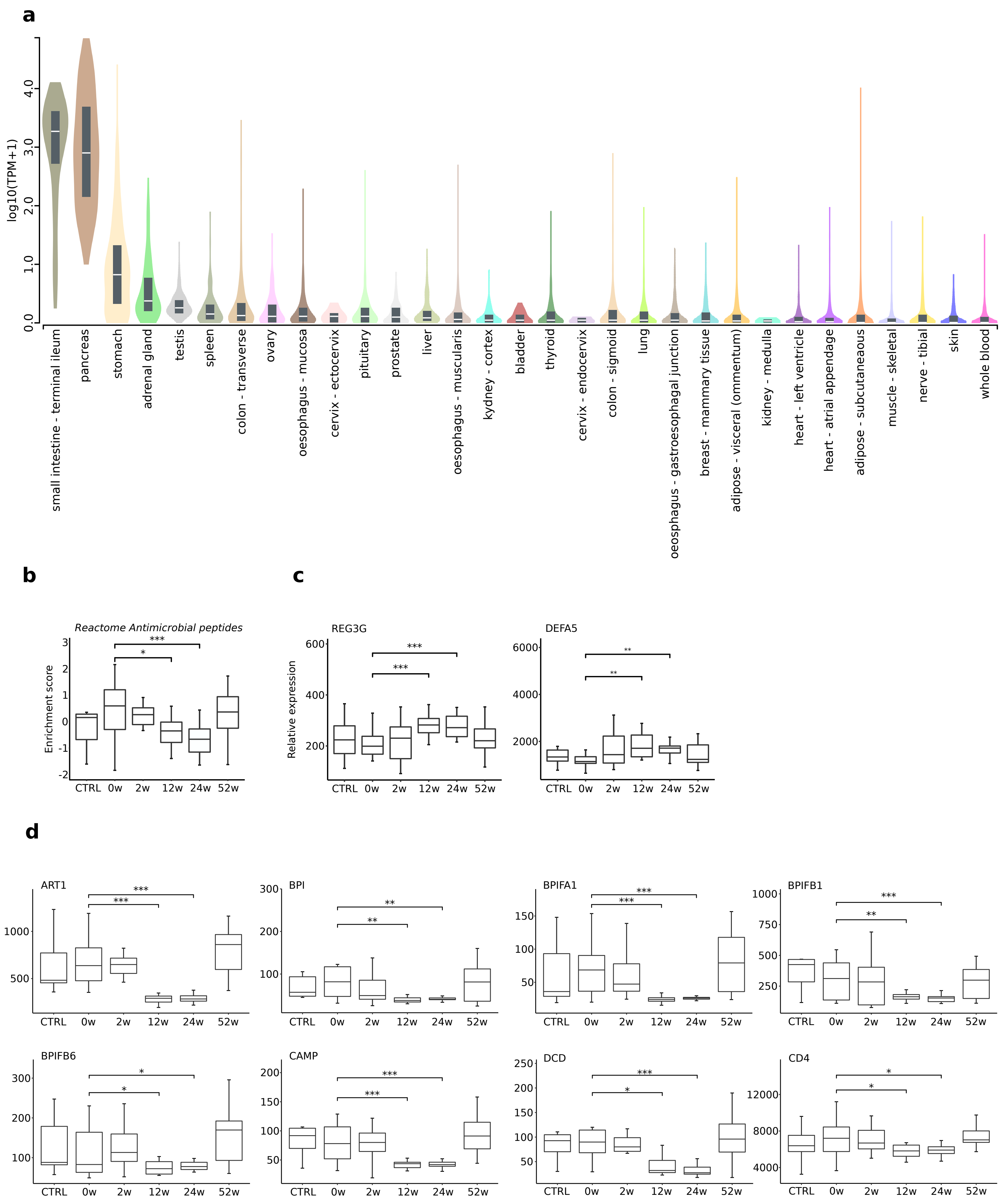

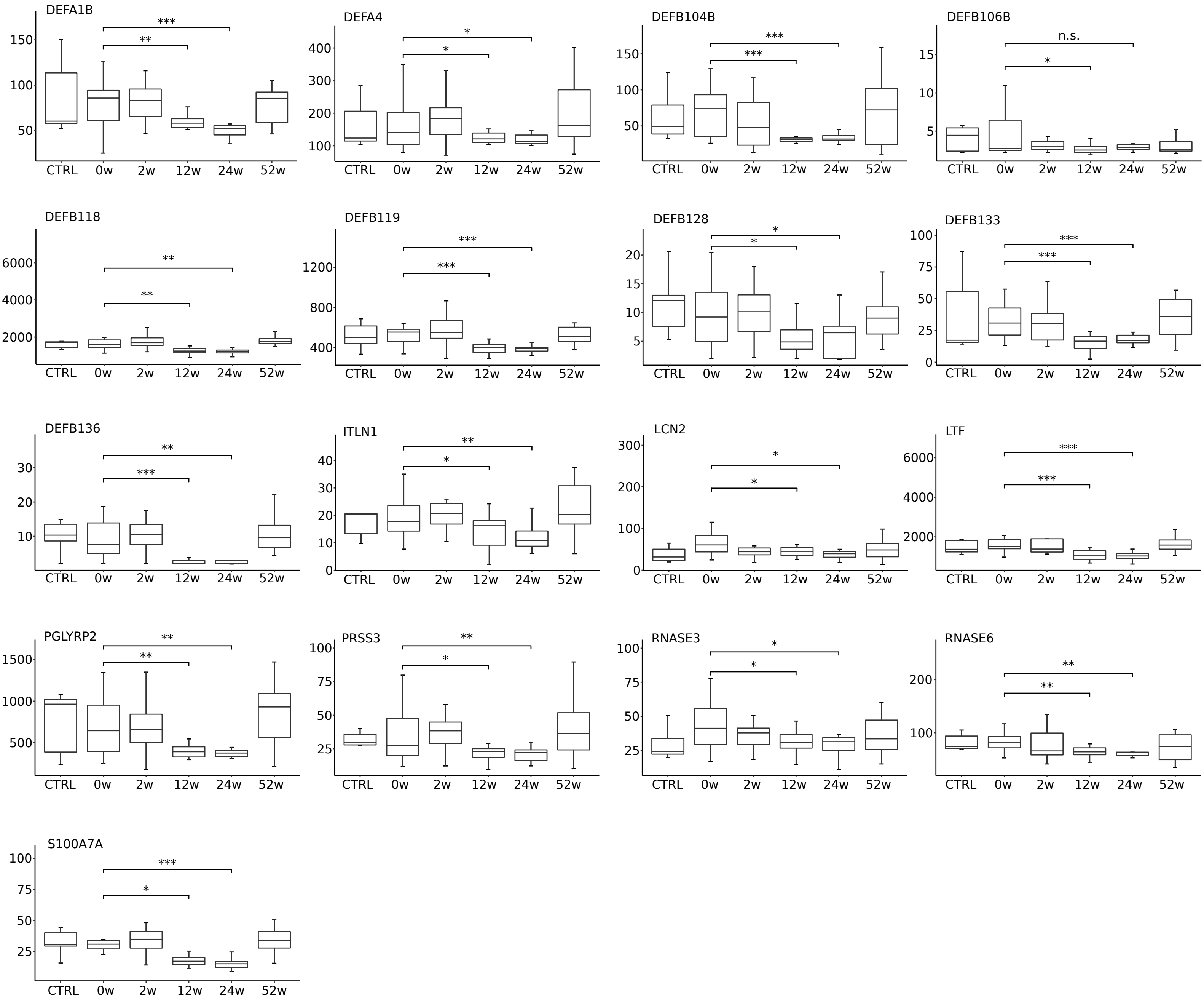
DEFA1B
150
100
50
***
**
CTRL 0w 2w 12w 24w 52w
DEFA4
400
300
200
100
*
*
CTRL 0w 2w 12w 24w 52w
DEFB104B
150
100
50
***
***
CTRL 0w 2w 12w 24w 52w
DEFB106B
15
10
5
n.s.
*
CTRL 0w 2w 12w 24w 52w
DEFB118
6000
4000
2000
**
**
CTRL 0w 2w 12w 24w 52w
DEFB119
1200
800
400
***
***
CTRL 0w 2w 12w 24w 52w
DEFB128
20
15
10
5
*
*
CTRL 0w 2w 12w 24w 52w
DEFB133
100
75
50
25
0
***
***
CTRL 0w 2w 12w 24w 52w
DEFB136
30
20
10
**
***
CTRL 0w 2w 12w 24w 52w
ITLN1
40
30
20
10
0
**
*
CTRL 0w 2w 12w 24w 52w
LCN2
300
200
100
0
*
*
CTRL 0w 2w 12w 24w 52w
LTF
6000
4000
2000
***
***
CTRL 0w 2w 12w 24w 52w
PGLYRP2
1500
1000
500
**
**
CTRL 0w 2w 12w 24w 52w
PRSS3
100
75
50
25
**
*
CTRL 0w 2w 12w 24w 52w
RNASE3
100
75
50
25
*
*
CTRL 0w 2w 12w 24w 52w
RNASE6
200
100
**
**
CTRL 0w 2w 12w 24w 52w
S100A7A
100
75
50
25
***
*
CTRL 0w 2w 12w 24w 52w

Supplementary Figure 11

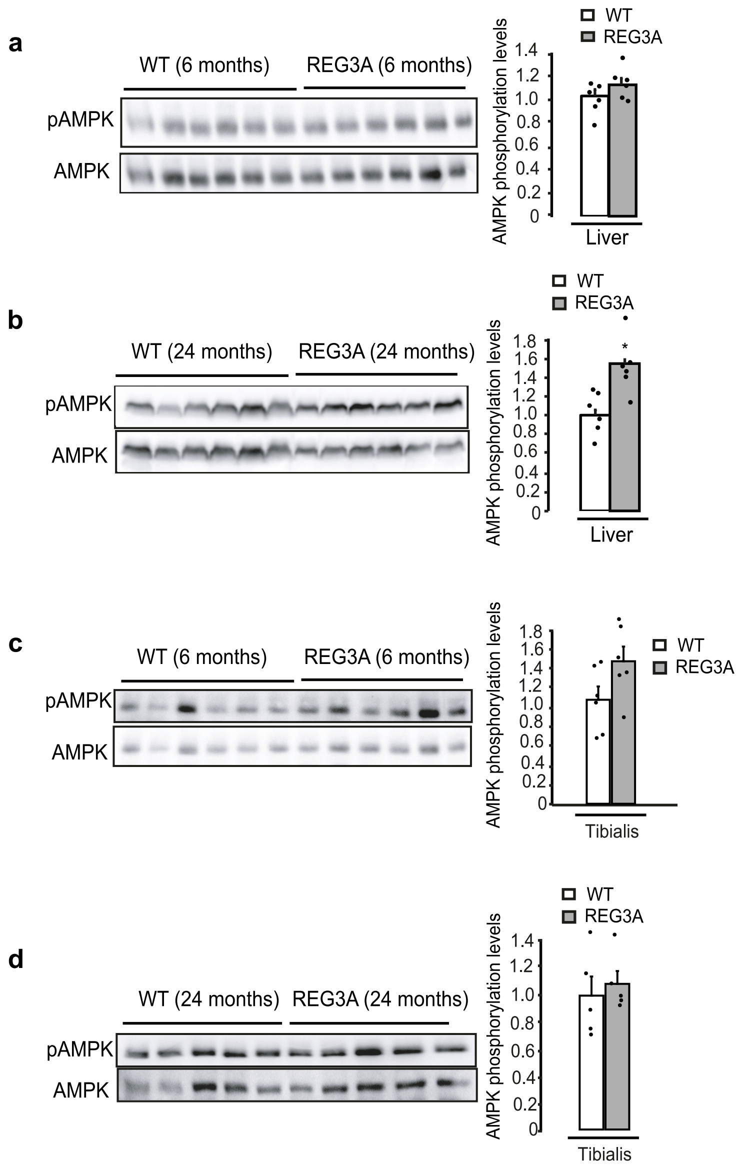

Gonzalez et al. Antimicrobial protein REG3A regulates glucose homeostasis and insulin resistance in obese diabetic mice

Supplementary Figures Legends

Supplementary Figure 1. Effects of the REG3A transgene on body weight of female mice. Changes in body weight over time in female REG3A transgenic mice (REG3A, n=75) and littermate wild-type (WT) controls (n=52) fed with ad libitum standard chow (2830 kcal/kg). $*p < 0.05$; $**p < 0.01$; $***p < 0.005$ by two-way repeated measures Anova followed by Tukey's post hoc multiple comparison test. NS, not significant.

Supplementary Figure 2. Metabolic parameters of 24-month-old REG3A transgenic and wild-type mice. (**a**) Cumulative food intake over a 3.5-day period. (**b**) Respiratory quotient (VCO2/VO2) during a day-night cycle. (**c**) Fatty acid (FA) oxidation measured by respirometry during a day-night cycle. REG3A: REG3A transgenic mice; WT: wild-type mice. n=8 for each group, same cohort as Figure 1. Data are averages ± SEM. $*p < 0.05$ by two-way repeated measures Anova followed by Tukey's post hoc multiple comparison test.

Supplementary Figure 3. Changes in phospho-Akt levels in REG3A transgenic mice fed a standard diet. (**a**) C-peptide concentrations in response to glucose intake in REG3A transgenic (REG3A) and wild-type (WT) mice at 6 and 24 months. **b**, **c** Immunoblots for Akt phosphorylation (Ser473) and total Akt protein levels in liver (**b**) and subcutaneous white adipose tissue (SCWAT), tibialis anterior and soleus muscles (**c**) of 6- and 24-month-old WT and REG3A mice fed a standard diet. Each lane represents one mouse. Bar chart: densitometry quantification.

Supplementary Figure 4: **Glucose homeostasis in female mice fed a high-fat diet**. (**a**) Body weight. REG3A: REG3A transgenic mice. WT: wild-type mice (n=4 for each group). CD: chow diet. HFD: high-fat diet. (**b**) Blood glucose curves under ITT insulin tolerance test. Right: area under the ITT curve. (**c**) Blood glucose curves in oral glucose tolerance tests OGTT. Right: area under the OGTT curve. Male groups correspond to the same cohort as in Figure 3. Data are means ± SEM. $*p<0.05$ by Anova followed by Tukey's post hoc multiple comparison test. No indication, no significance.

Supplementary Figure 5: Changes in phospho-Akt levels in REG3A transgenic mice fed a high-fat diet. Immunoblots for Akt phosphorylation (Ser473) and total Akt protein levels in the liver (**a**) tibialis (**b**) white adipose tissue (WAT; **c**) and soleus (**d**) of REG3A transgenic (REG3A) and control (WT) mice fed a high-fat diet. Each lane represents one mouse. Bar chart: densitometric analysis. The data are averages ± SEM. NS or no statistical indication, no significance (Student's t-test).

Supplementary Figure 6: Effect of REG3A on fat mass and fatty liver in obese ob/ob mice. Three-month-old ob/ob mice were given a daily subcutaneous dose of 9µg or 43µg of a recombinant human REG3A protein (rcREG3A) for 28 days (n=10 per group). Control ob/ob mice received an equivalent volume of buffer (vehicle). (**a**) Hepatic lipid levels. (**b**) Histological score of liver steatosis on day 25 after starting rcREG3A or buffer treatment.

Supplementary Figure 7. REG3A does not alter pancreatic islets under a high fat diet. Quantification of insulin-producing islet surface area in WT mice fed a high-fat diet that received 43µg per day of a recombinant REG3A protein (rcREG3A) subcutaneously or an equivalent volume of buffer (vehicle) for 28 days. The data are averages ± SEM. NS, no significance (Student's t-test).

Supplementary Figure 8. Changes in phospho-Akt levels in recombinant REG3A-treated prediabetic mice. (**a**) Immunoblots for Akt phosphorylation (Ser473) and total Akt protein levels in tibialis anterior muscle. (**b**) Immunoblots for Akt phosphorylation (Ser473) and total Akt protein levels and Anti-phospho-AMPK (pAMPK) immunoblotting in the liver of prediabetic mice fed a high-fat diet that were subcutaneously administered recombinant human REG3A protein (rcREG3A) or buffer (vehicle) for 28 days. Each lane represents a single mouse.

Supplementary Figure 9. No change in proinflammatory cytokine levels in response to high-fat diet or leptin deficiency. Measurement of plasma cytokines (**a**) IL6. (**b**) TNF-α. (**c**) IFN-γ in REG3A transgenic (REG3A) and control (WT) mice fed a chow diet (CD) or a high-fat diet (HF260), in prediabetic mice fed a high-fat diet (HF235) and in ob/ob mice given a recombinant REG3A protein (rcREG3A) or buffer (vehicle). Each dot represents one mouse.

Supplementary Figure 10. Dynamic changes in antimicrobial peptide expression in muscle after bariatric surgery. (**a**) REG3A gene expression in indicated human tissues assessed from the GTEx portal. TPM: Transcripts per million. **b-d** Molecular changes over time in vastus lateralis skeletal muscle from 16 bariatric surgery patients (GSE 135066). (**b**) Gene enrichment of "antimicrobial peptides" in Reactome. (**c**) Right: mRNA expression of defensin A5 (DEFA5). Left: mRNA expression of REG3G. (**d**) Expression of indicated antimicrobial peptides. CTRL: healthy controls.

Supplementary Figure 11. Changes in phospho-AMPK levels in REG3A transgenic mice. Anti-phospho-AMPK (pAMPK) immunoblotting in liver (**a**, **b**) and tibialis anterior muscle (**c**, **d**) of 6- and 24-month-old REG3A transgenic mice (REG3A) and wild-type (WT) mice. Bar chart: densitometric analysis. Data are averages ± SEM. $*p < 0.05$ (Student's t test). No statistical indication, no significance.

Gonzalez et al. Antimicrobial protein REG3A regulates glucose homeostasis and insulin resistance in obese diabetic mice

Figure 6c

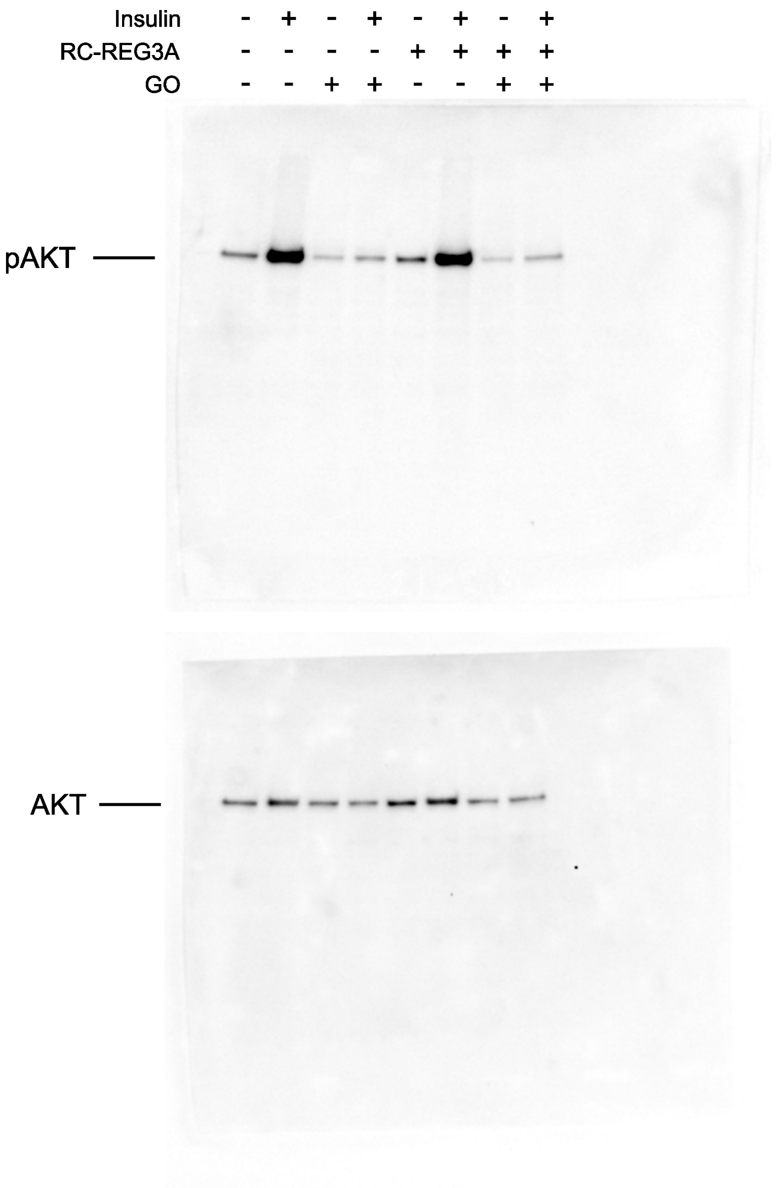

Figure 6d

pAMPK

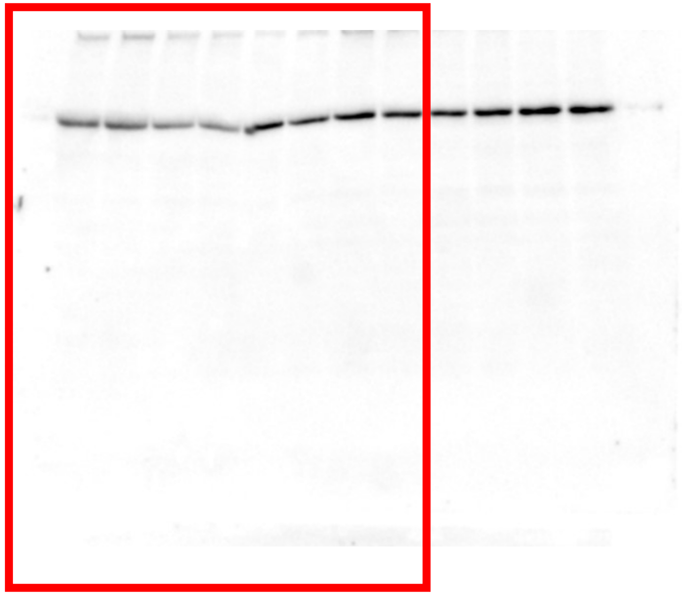

pAMPK
(visible)

75
50

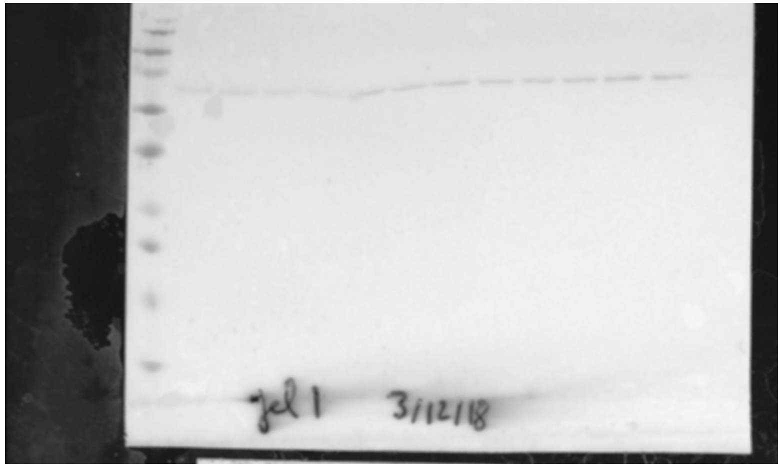

AMPK

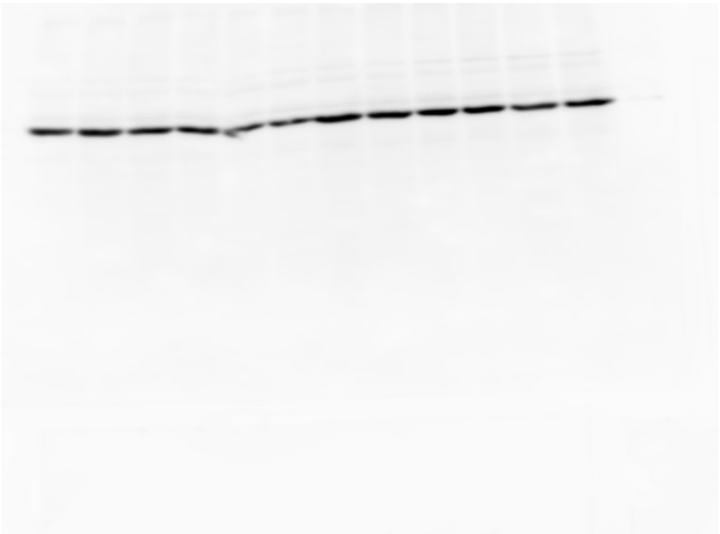

AMPK (visible)

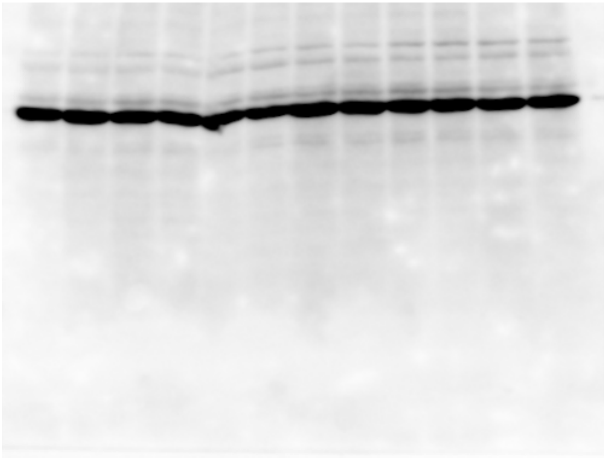

Figure 6e

pAMPK

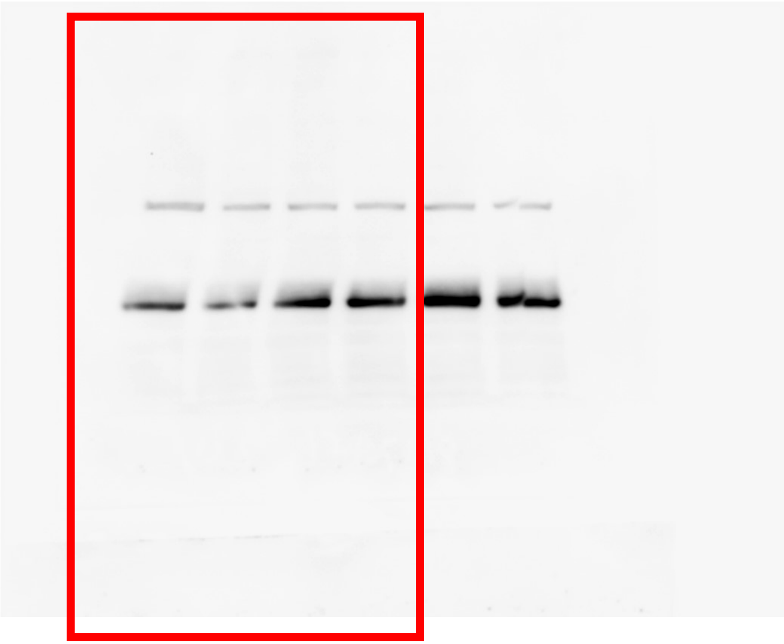

pAMPK
(visible)

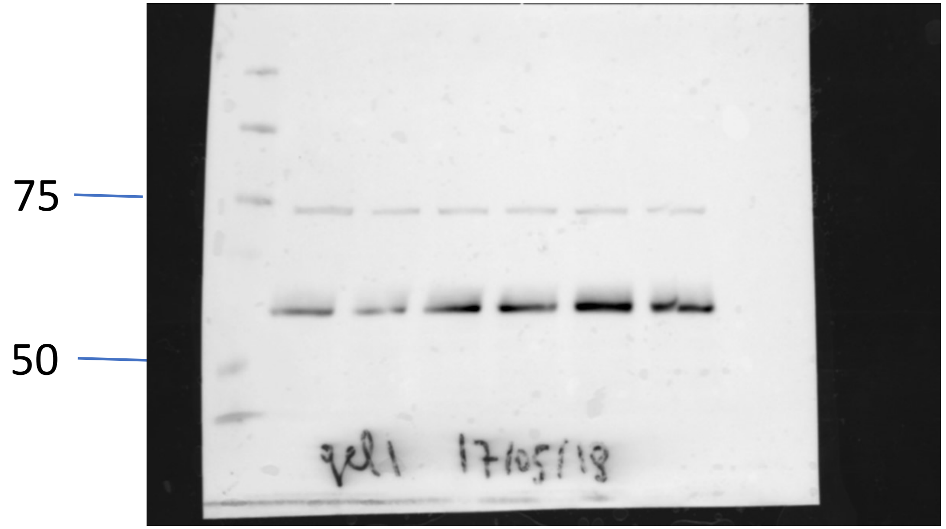

AMPK

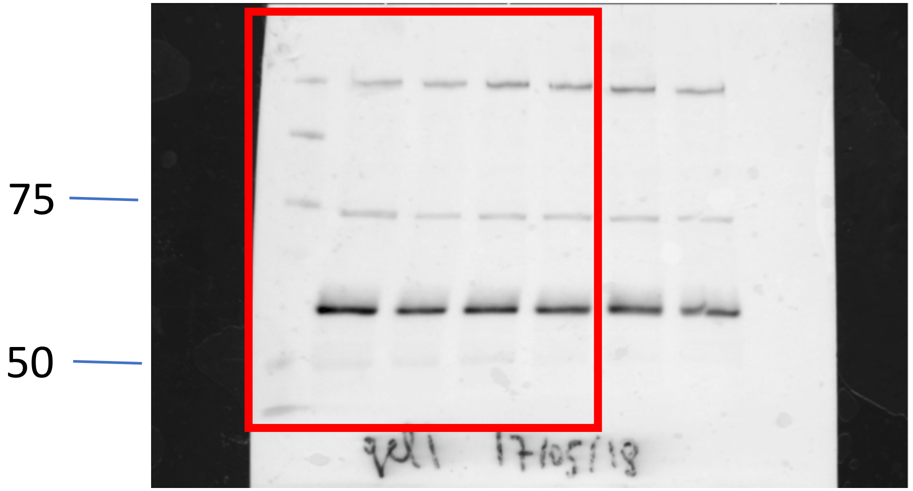

Figure 6f

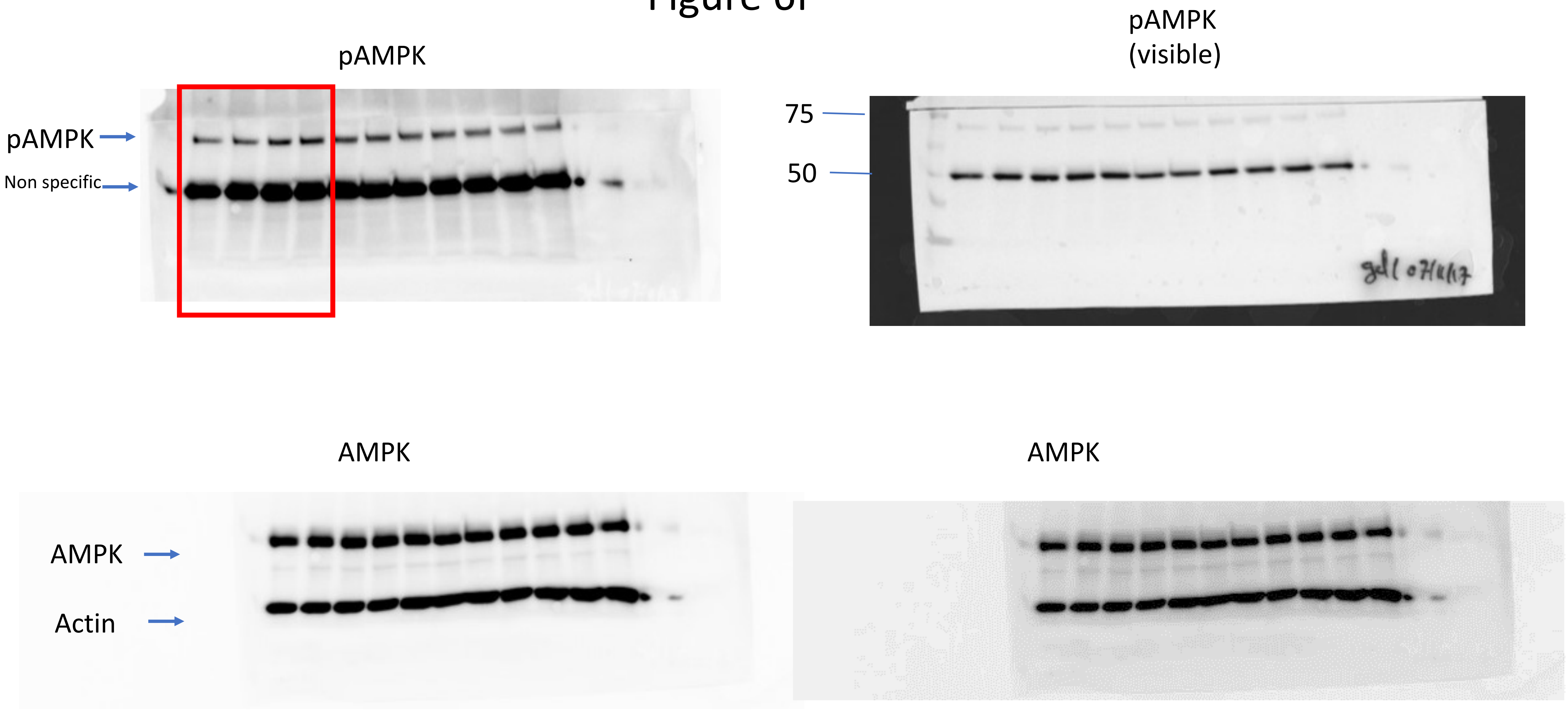
pAMPK
pAMPK (visible)
pAMPK
Non specific
75
50
AMPK
AMPK
AMPK
Actin

Figure 6g

pAMPK

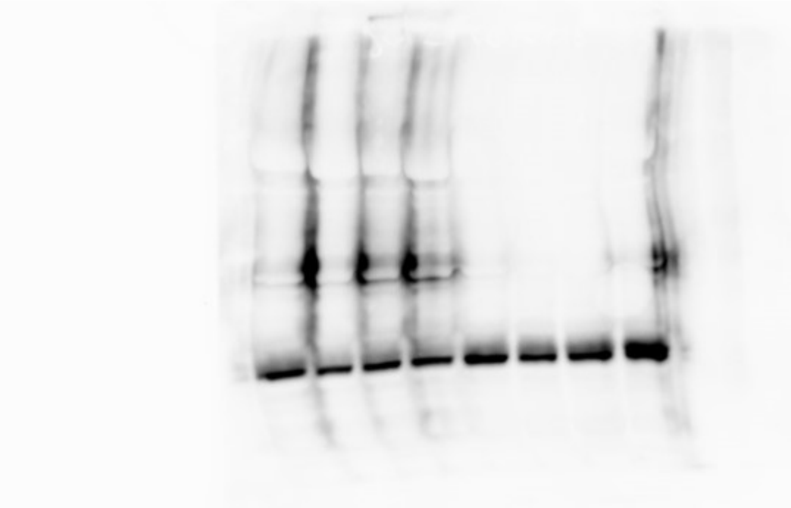

pAMPK
(visible)

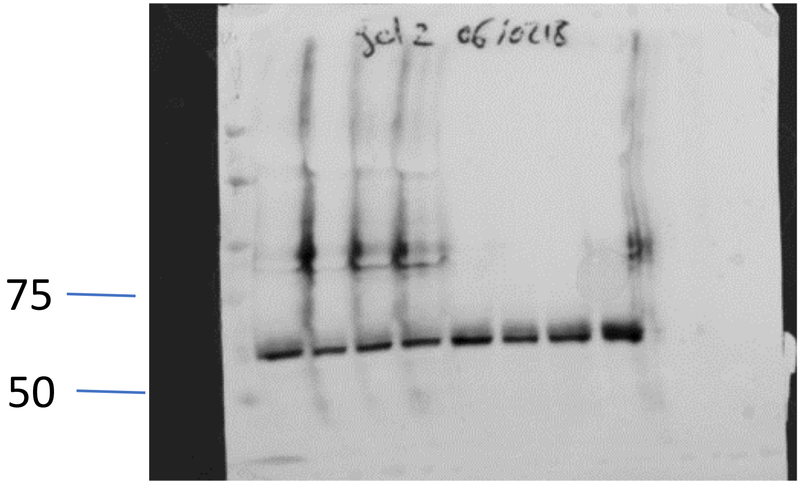

AMPK

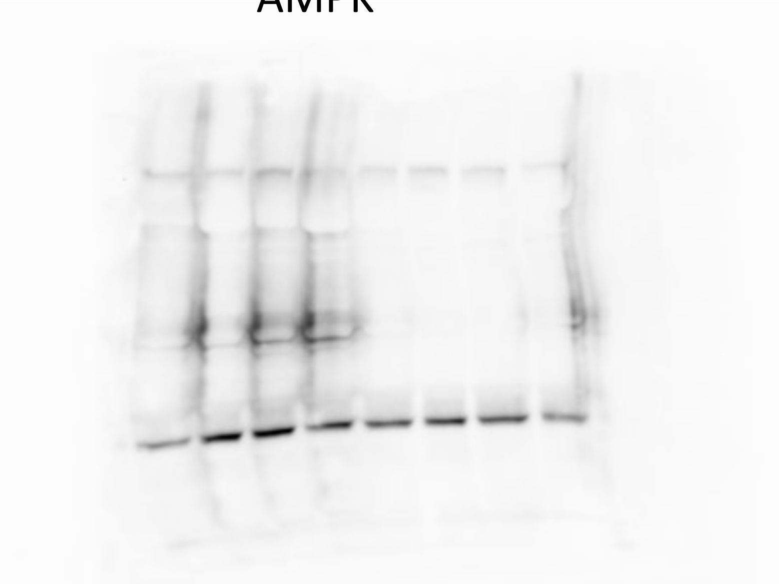

AMPK
(visible)

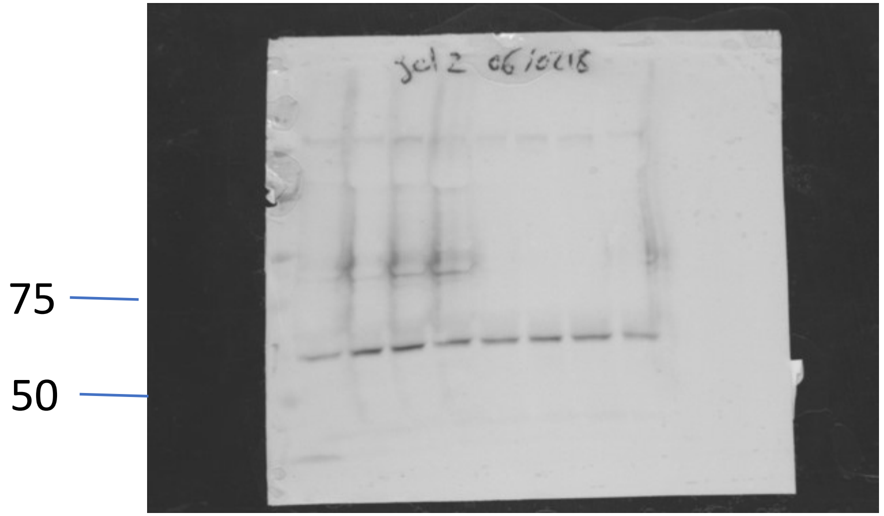

Figure 6g

pAKT

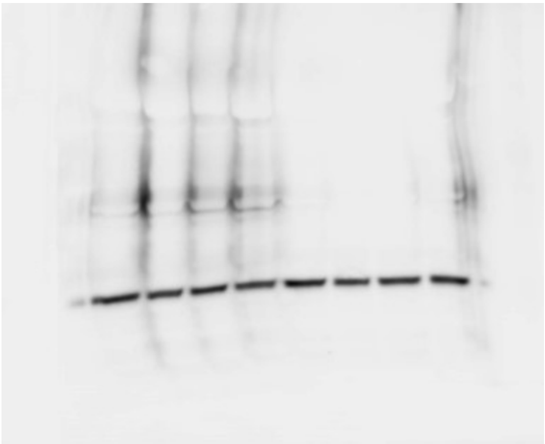

pAKT
(visible)

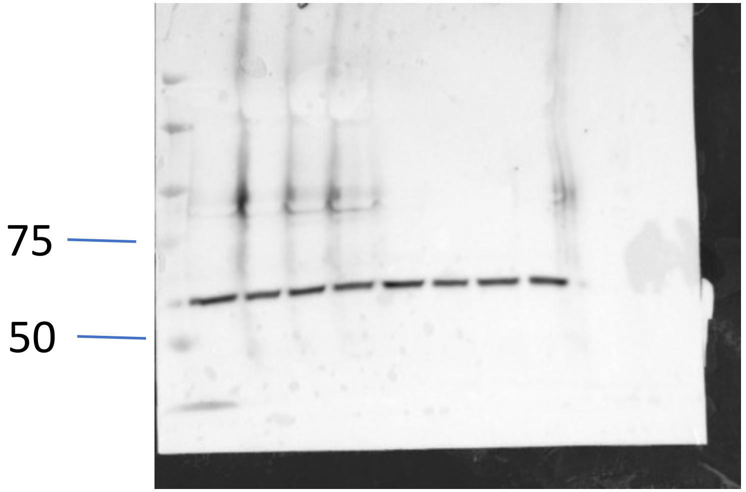

AKT

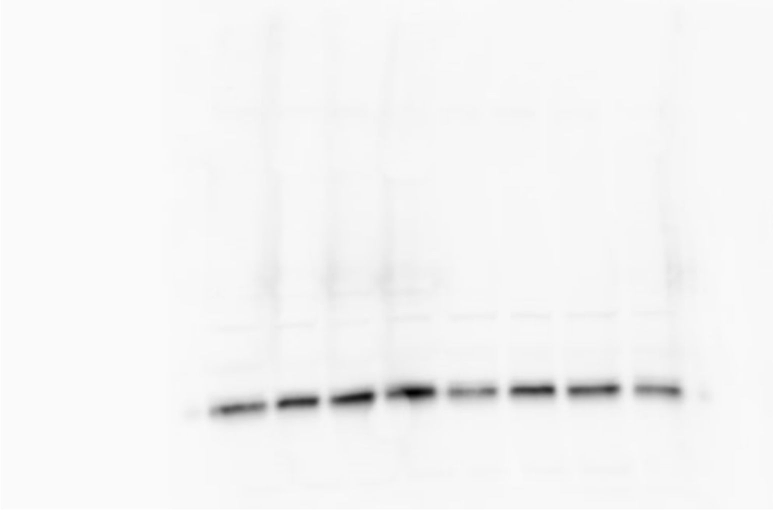

AKT
(visible)

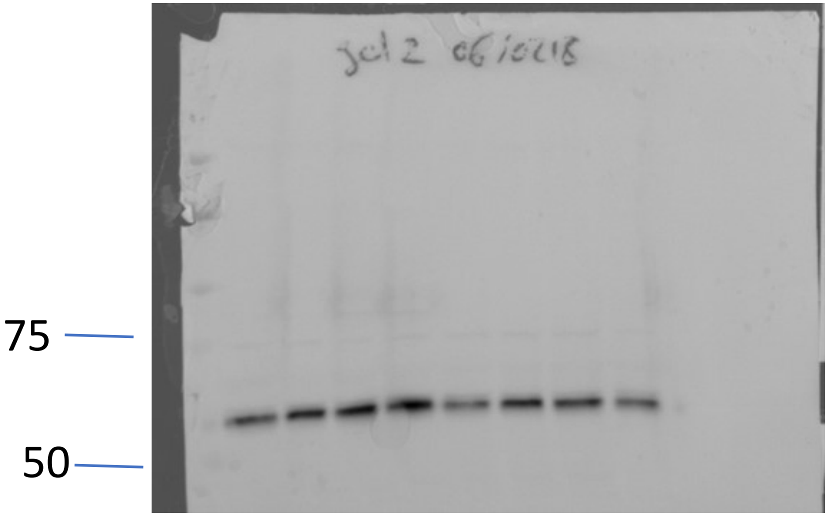

Figure 6h

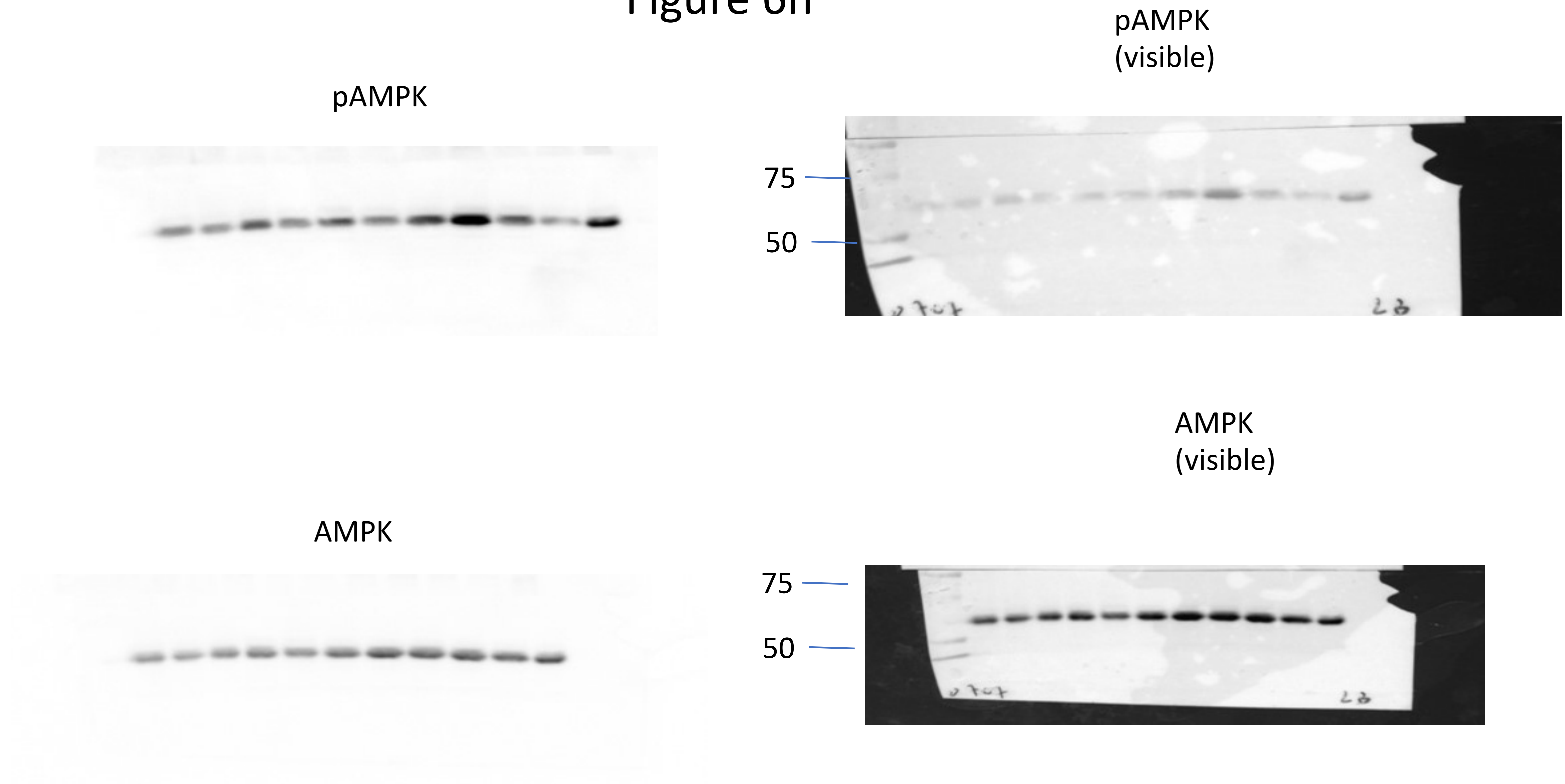

Figure 6h

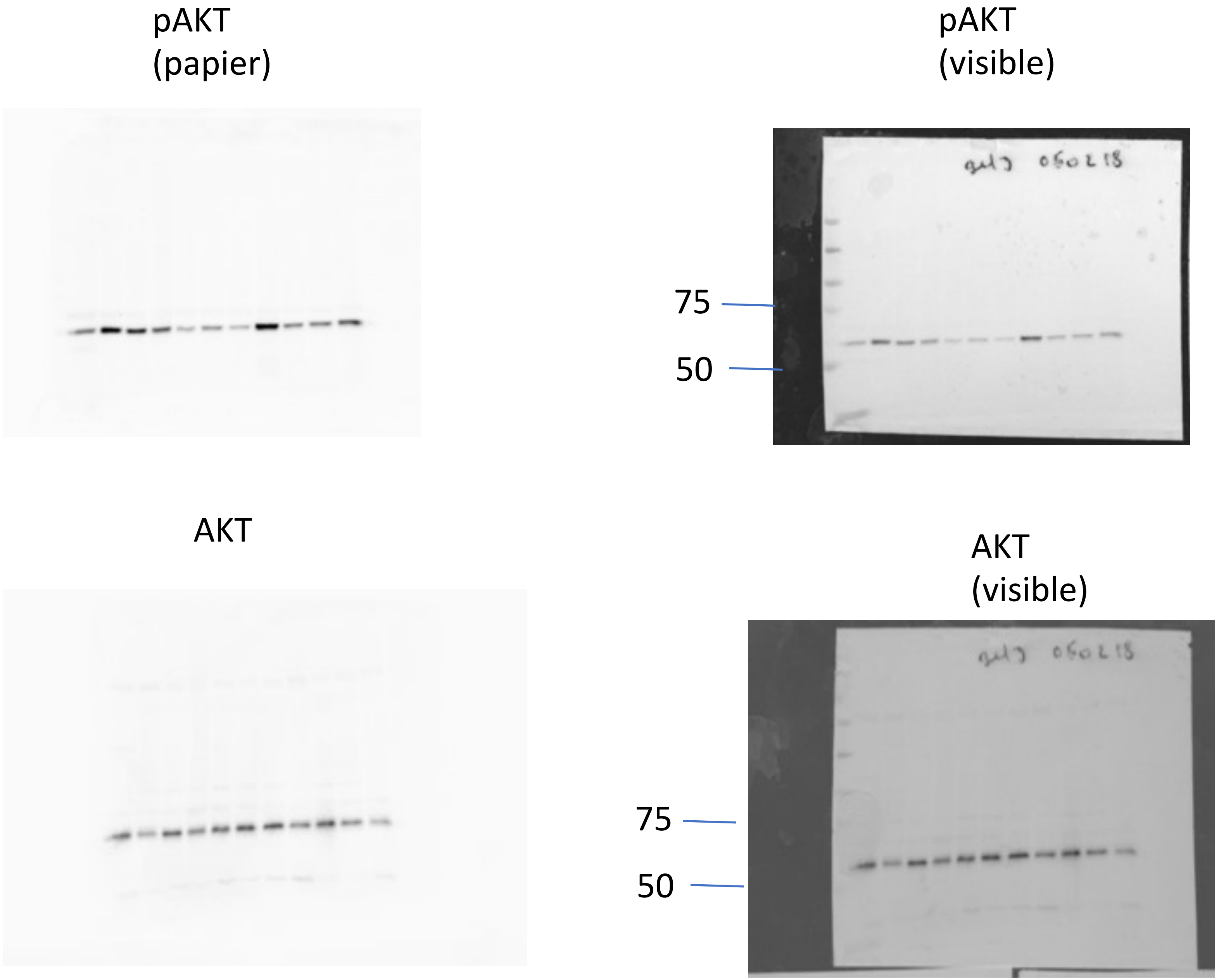

Figure 6i

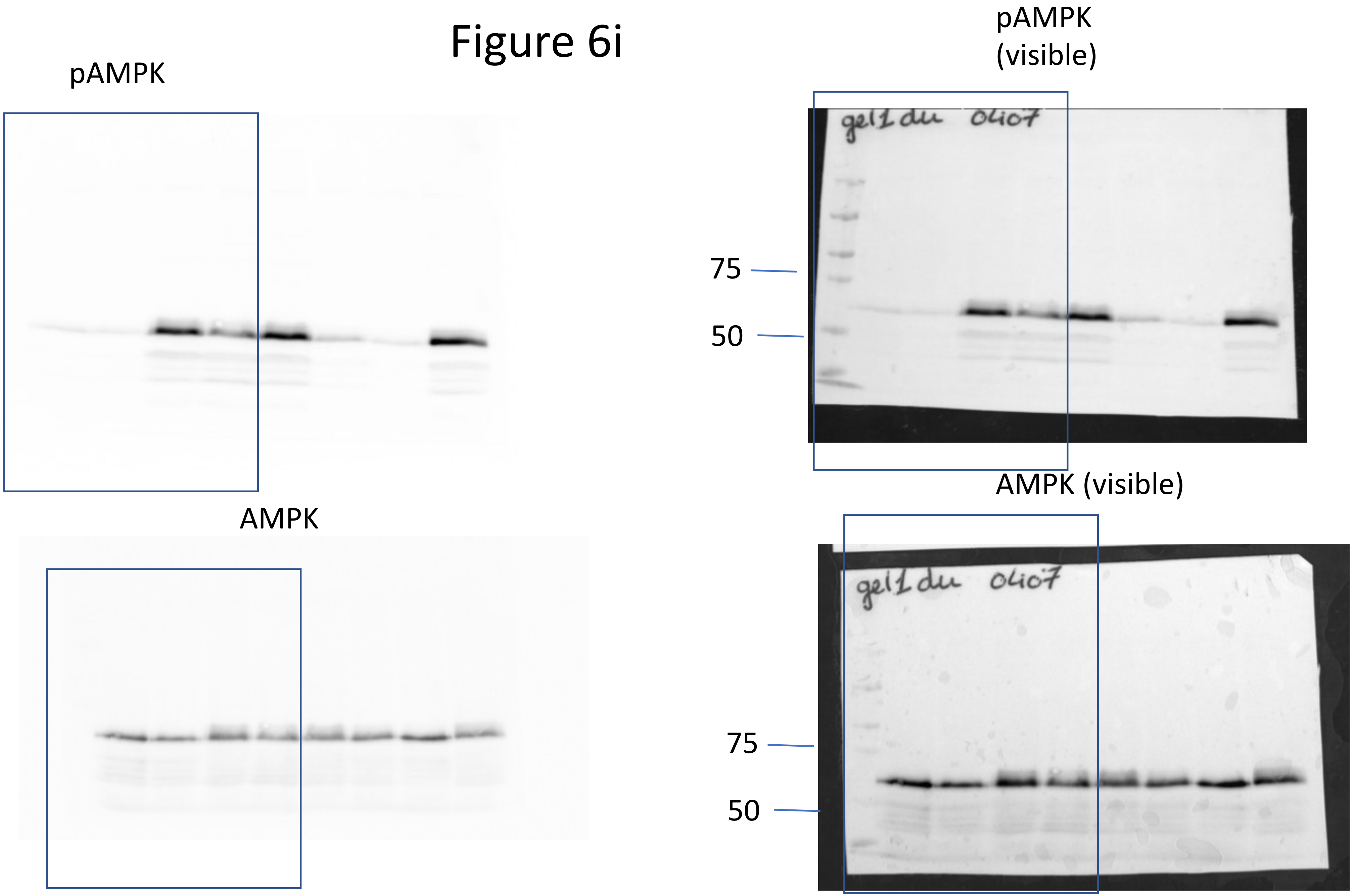

Figure 6i

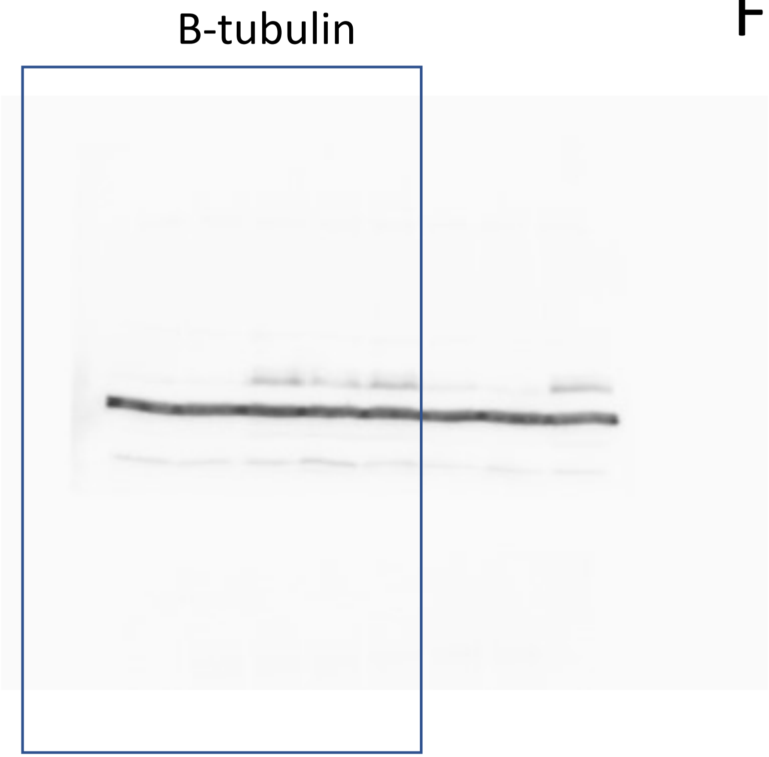

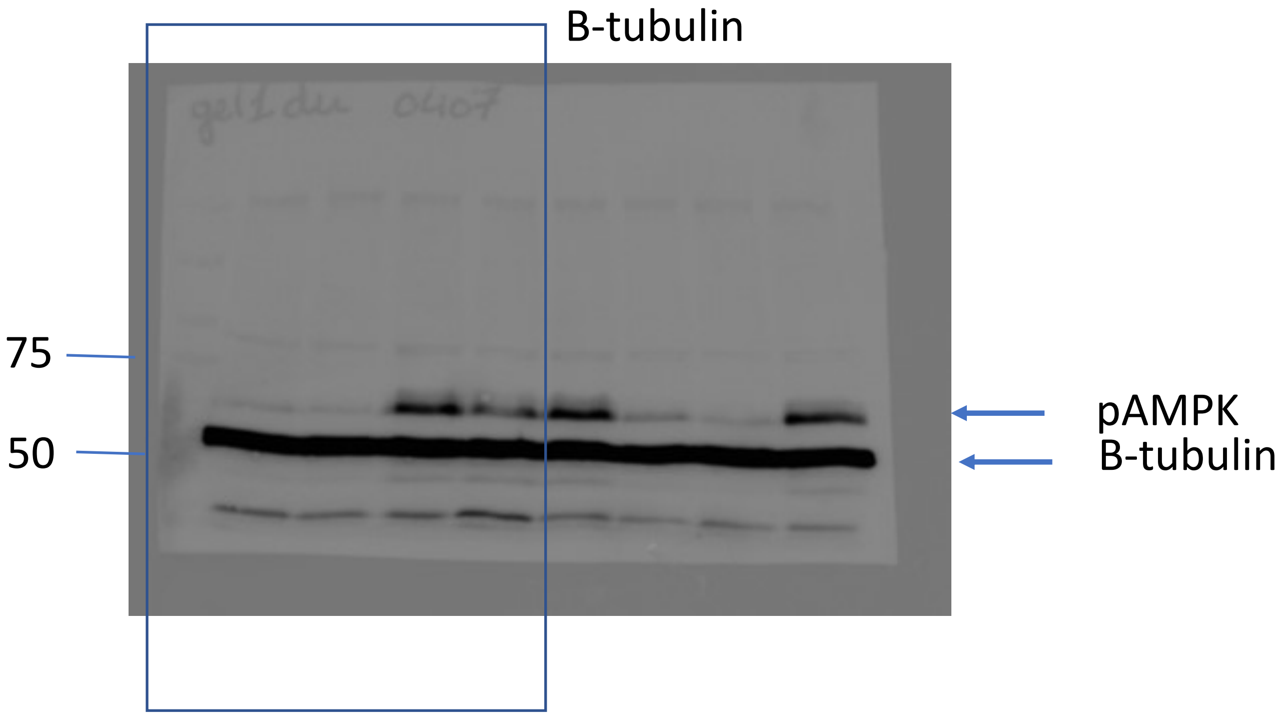

Figure 6j

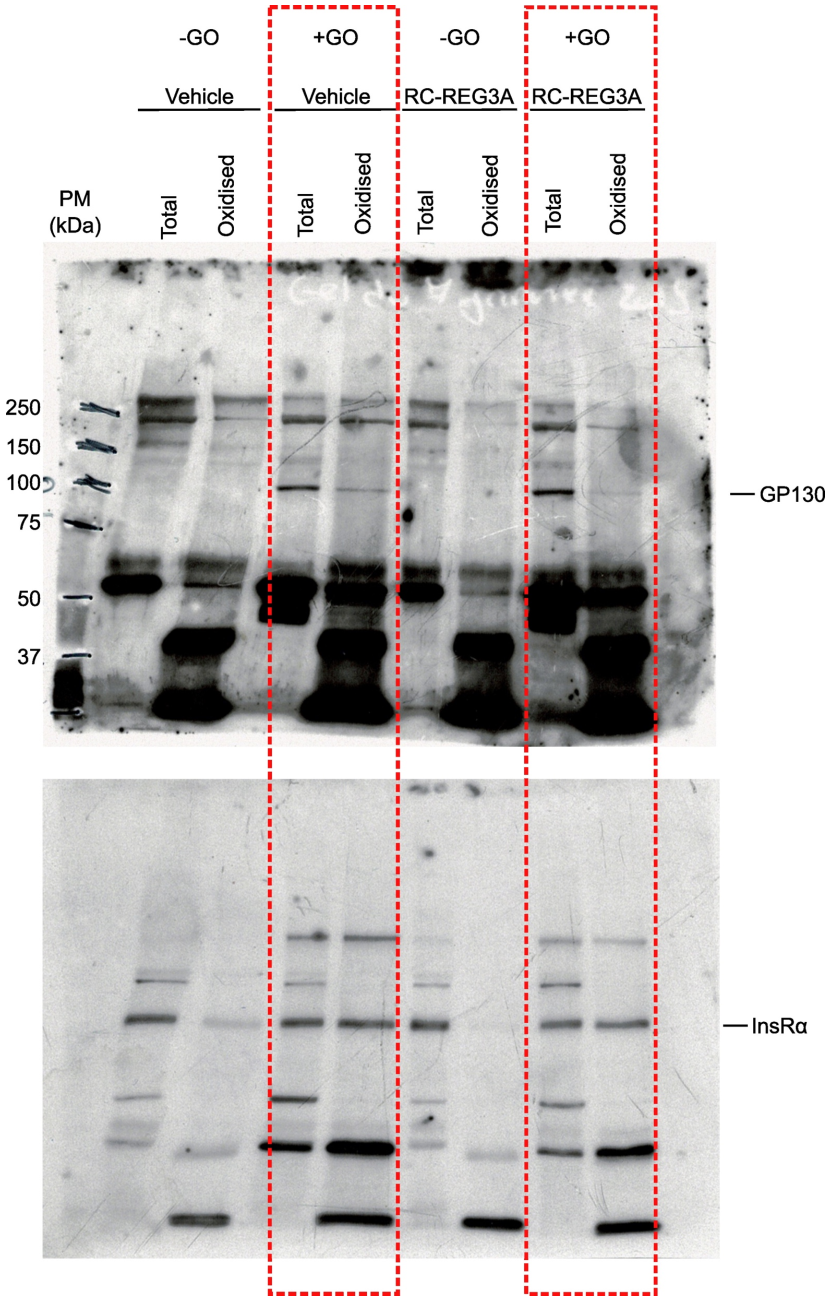

-GO
+GO
-GO
+GO
Vehicle
Vehicle
RC-REG3A
RC-REG3A
PM
(kDa)
Total
Oxidised
Total
Oxidised
Total
Oxidised
Total
Oxidised
250
150
100
75
50
37
GP130
InsRα

Figure S3b

pAKT

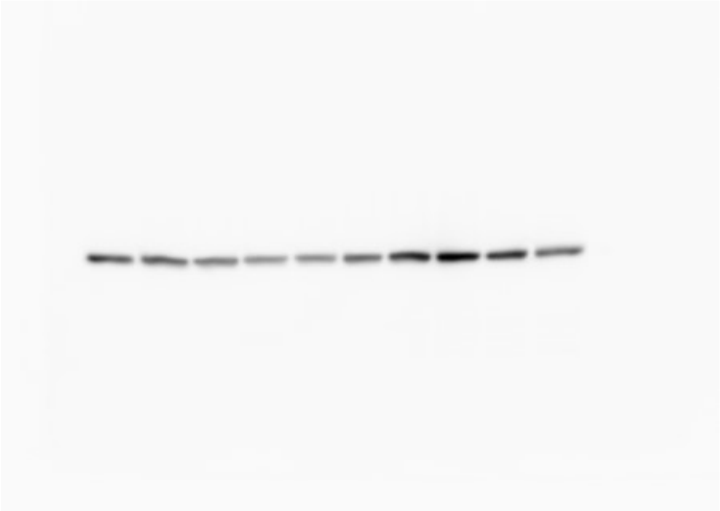

AKT

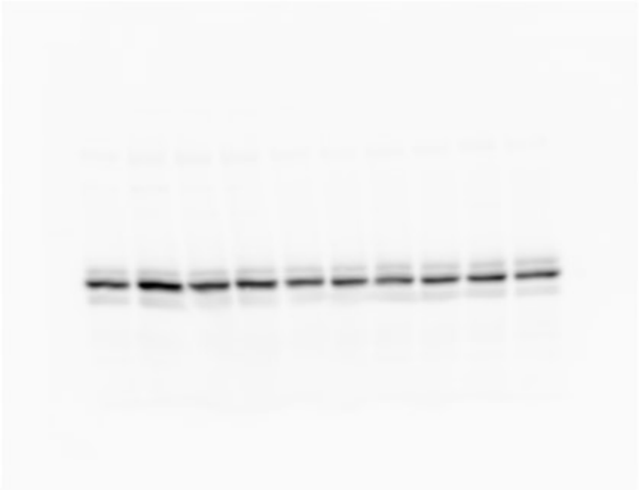

AKT
(visible)

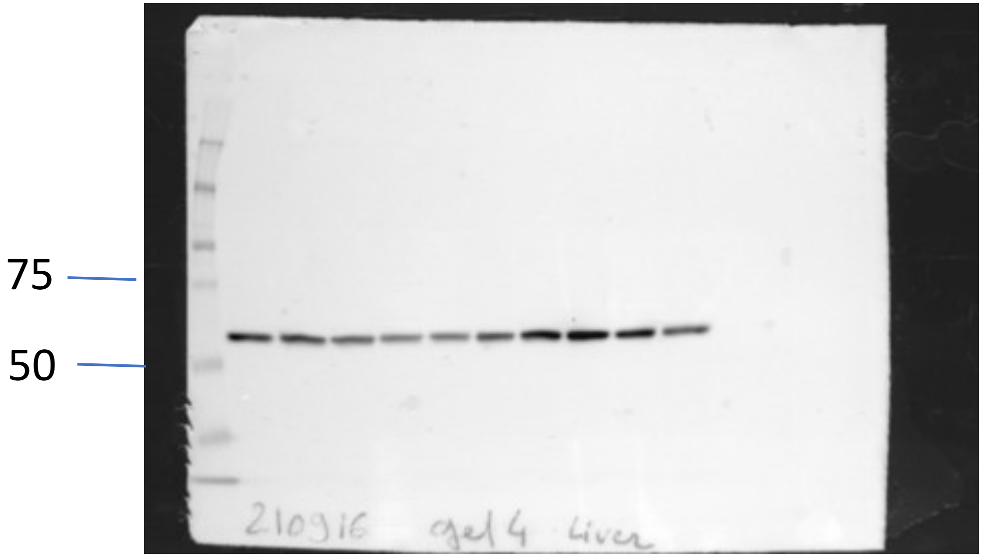

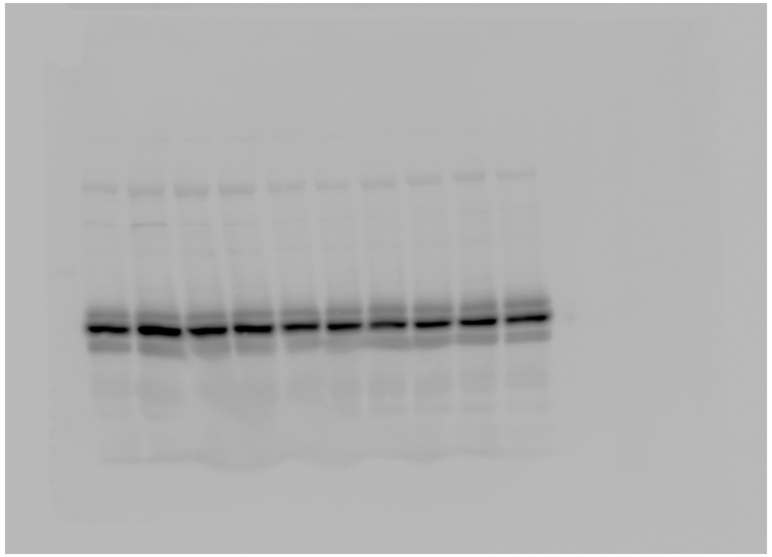

Figure S3b

pAKT

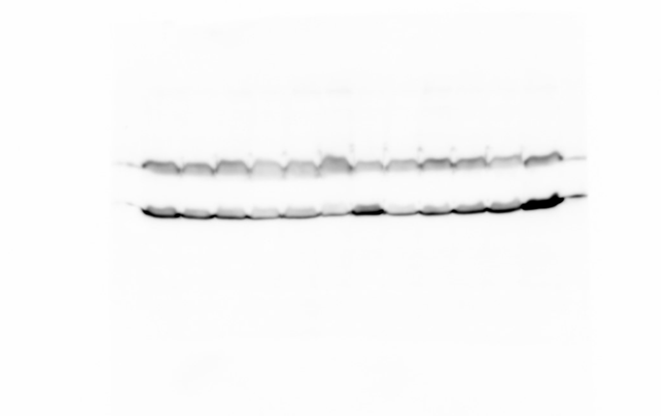

pAKT
(visible)

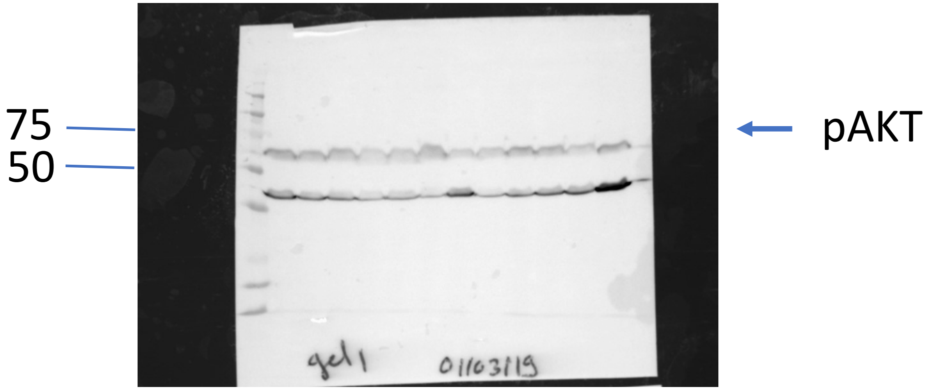

AKT

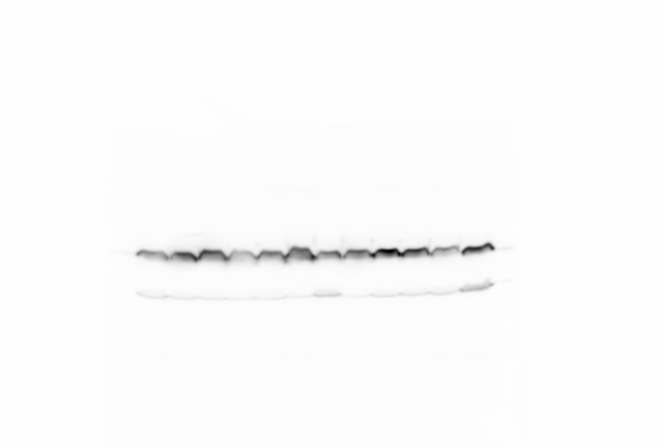

AKT
(visible)

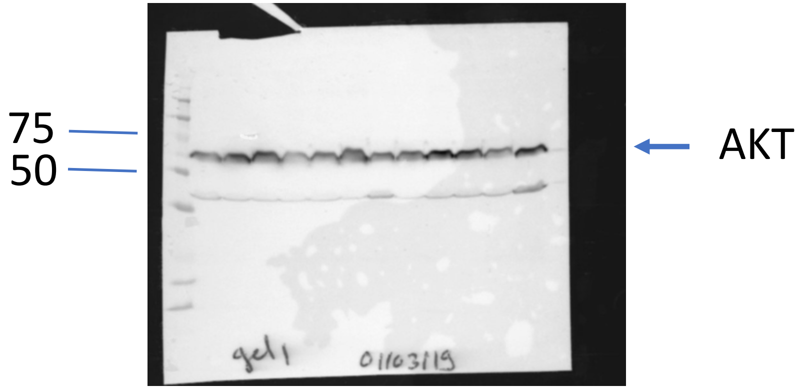

Figure S3c

pAKT

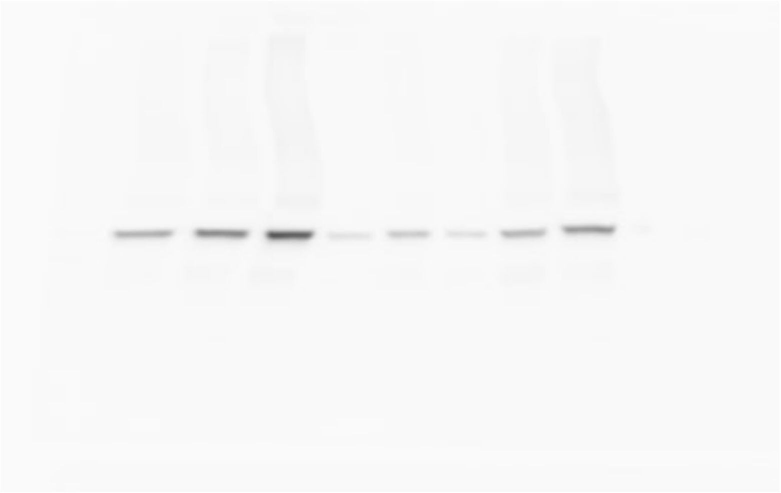

pAKT
(visible)

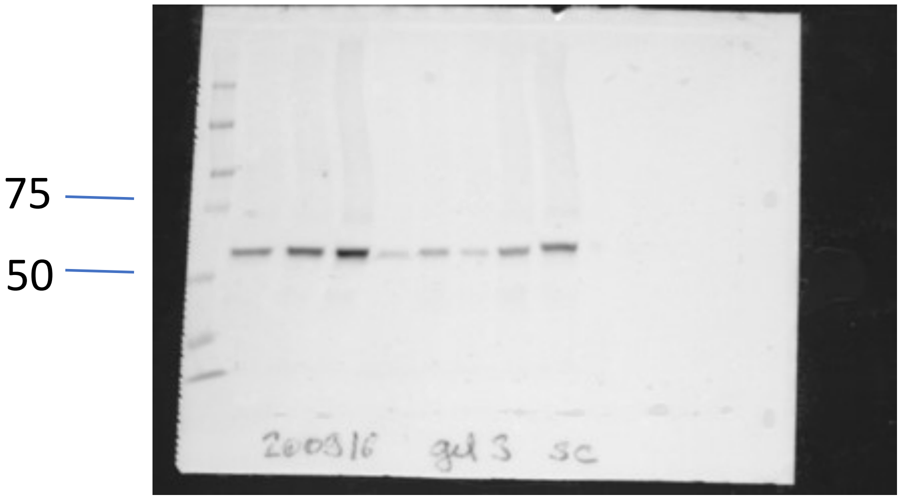

AKT

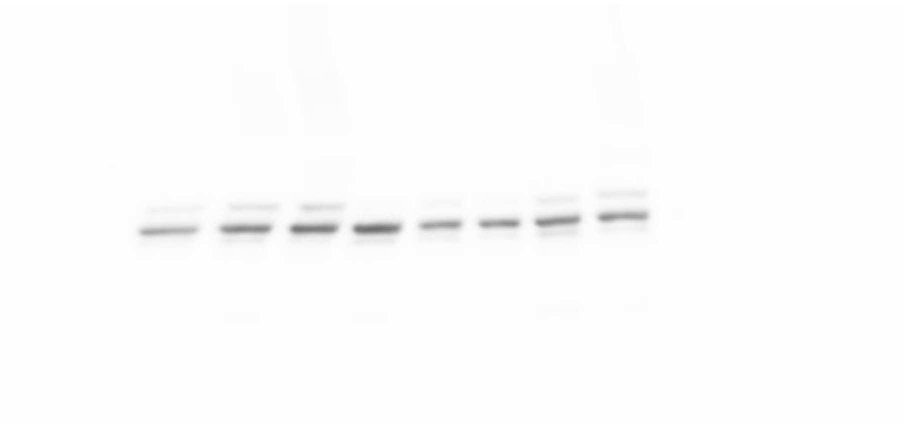

AKT
(visible)

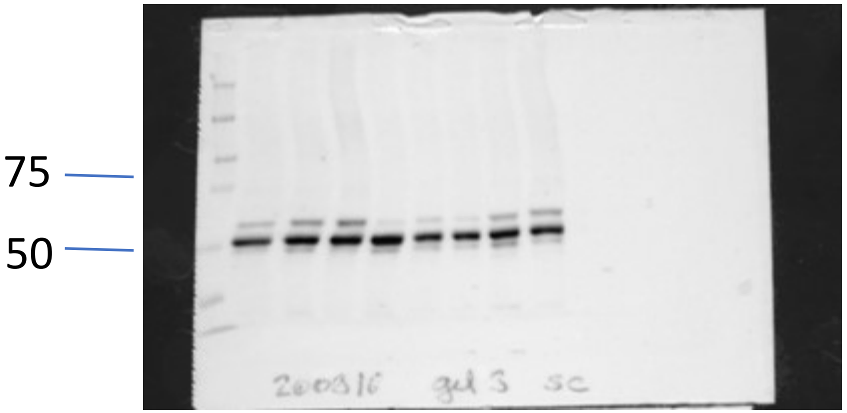

Figure S3c

pAKT

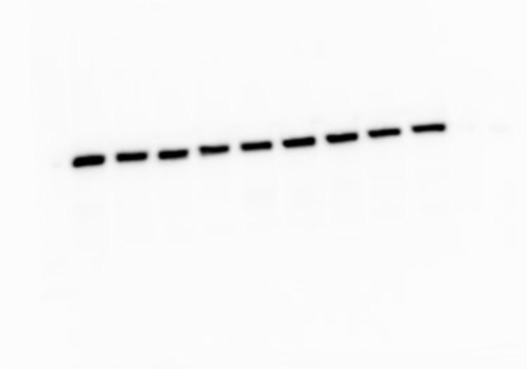

pAKT
(visible)

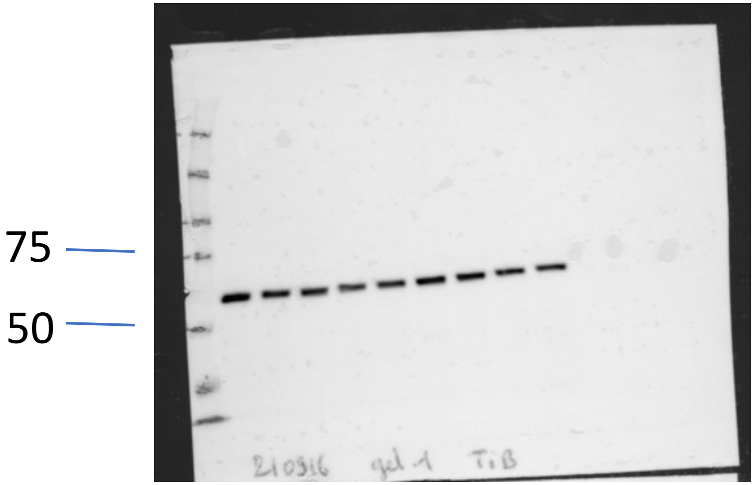

AKT

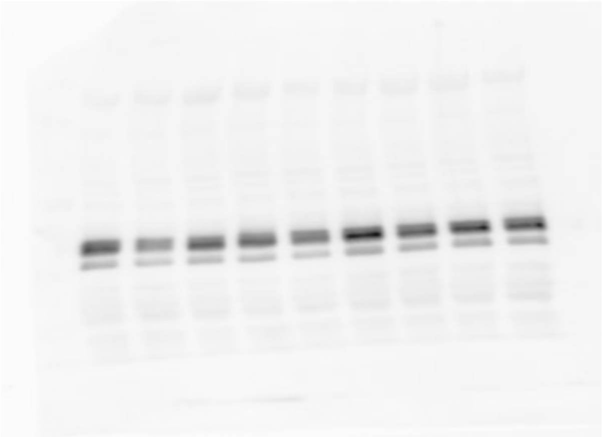

AKT
(visible)

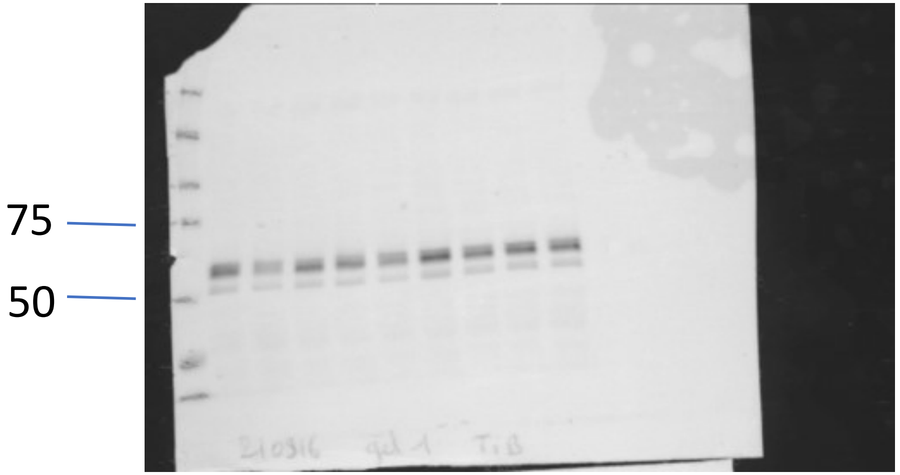

Figure S3c

pAKT

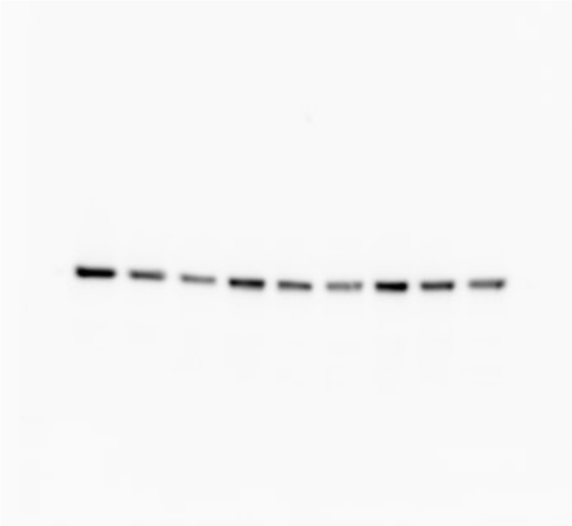

pAKT
(visible)

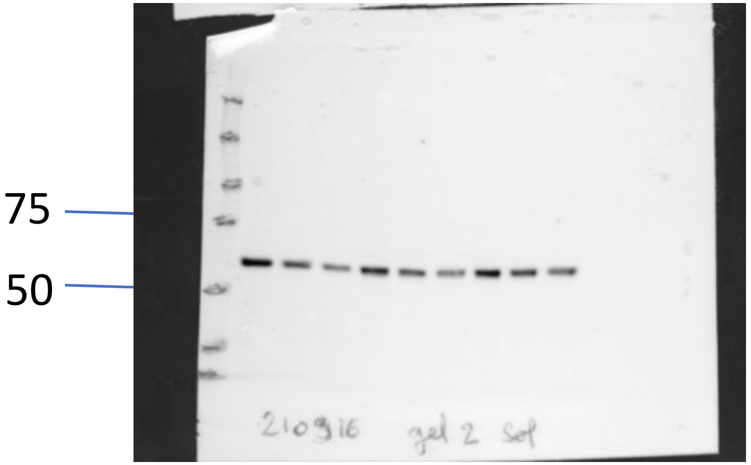

AKT

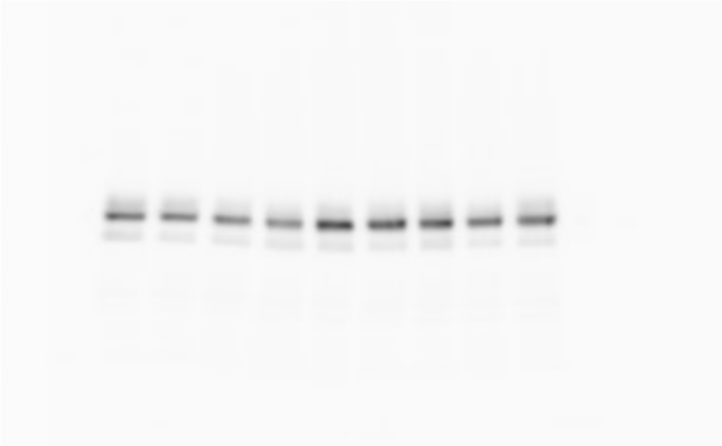

AKT
(visible)

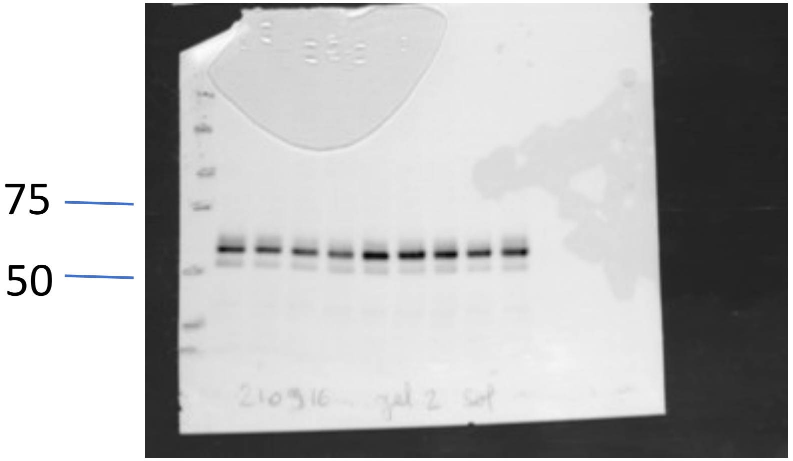

Figure S5a

pAKT

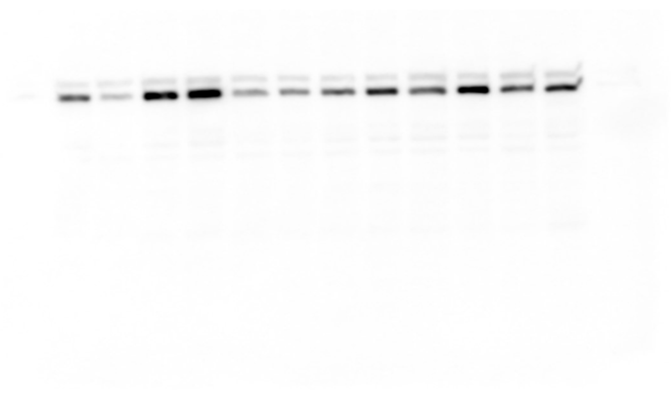

pAKT (visible)

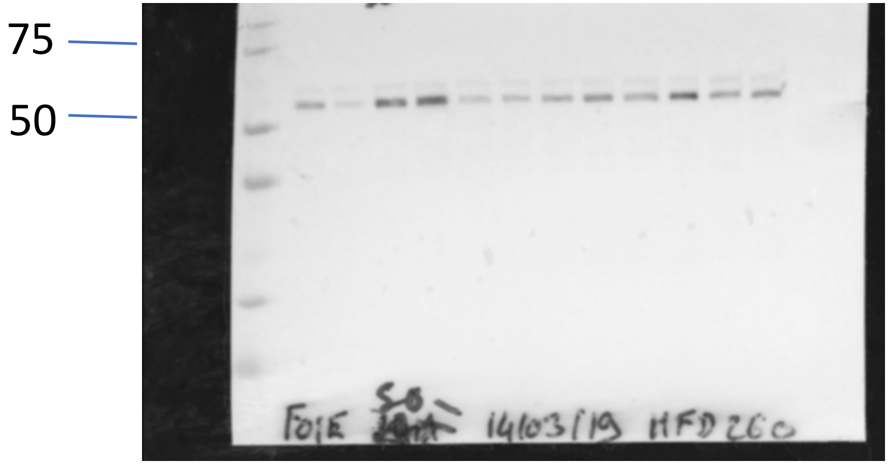

AKT (visible)

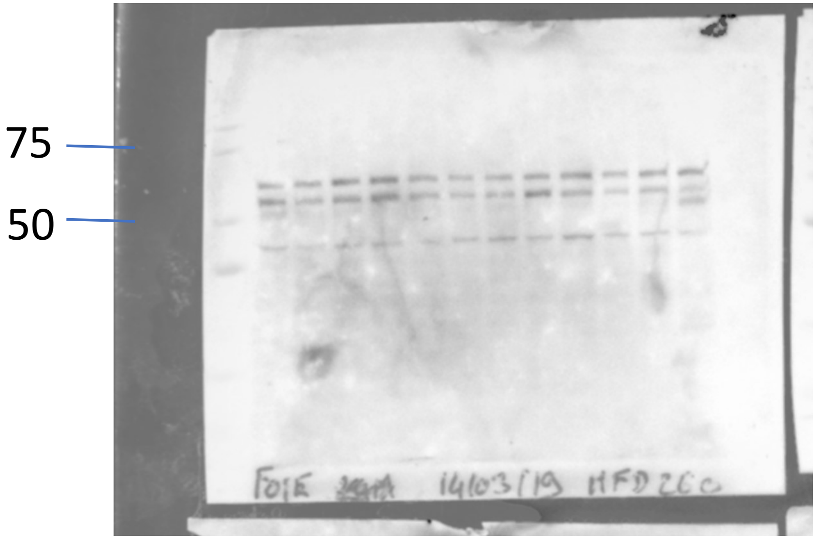

Figure S5b

pAKT

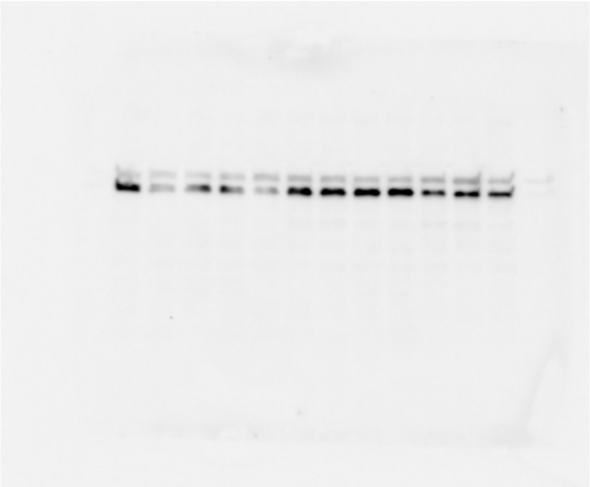

pAKT (visible)

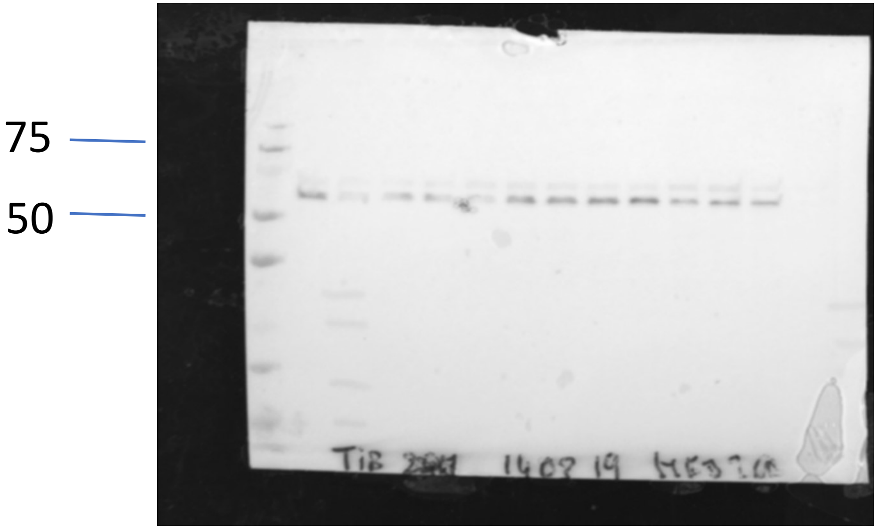

AKT

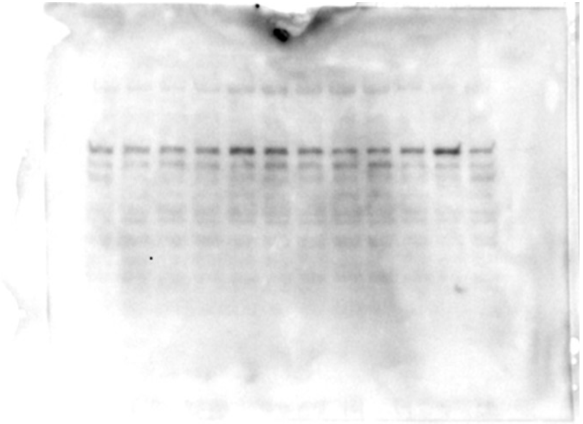

AKT (visible)

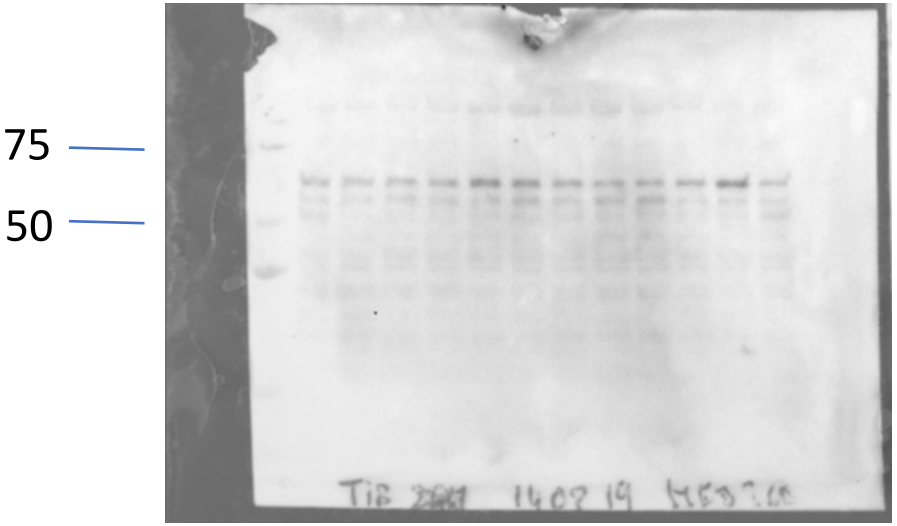

Figure S5c

pAKT

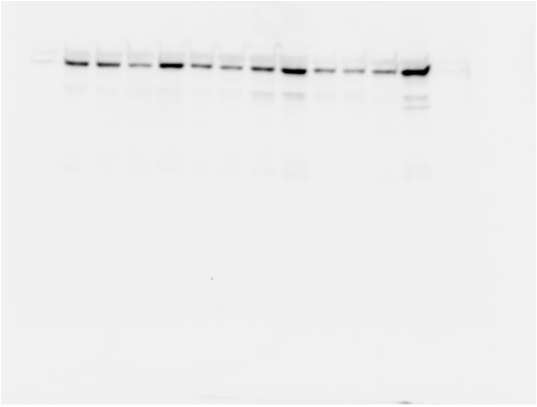

pAKT
(visible)

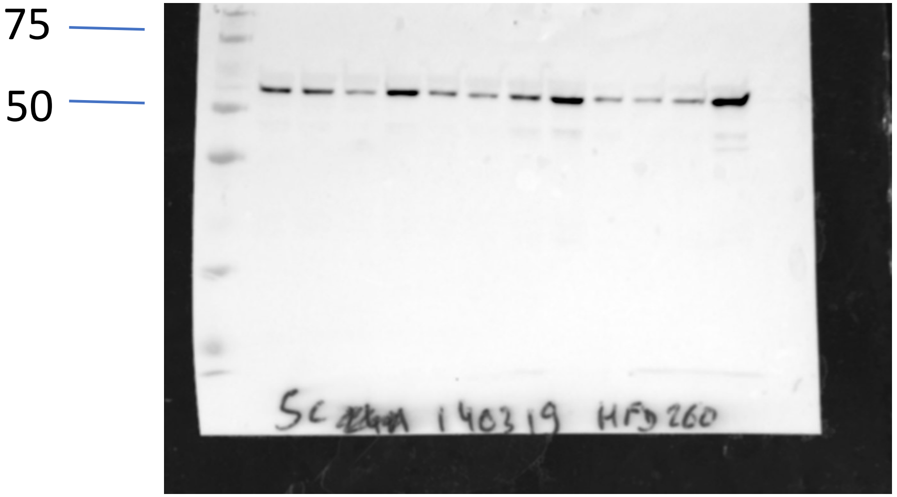

AKT

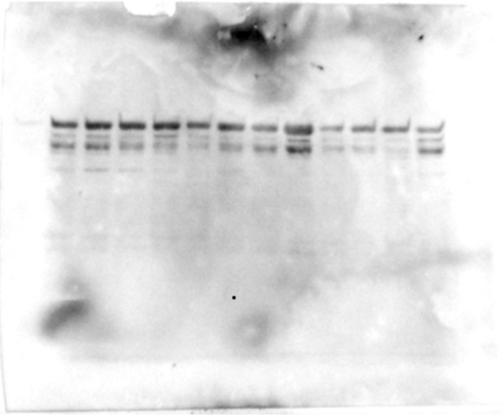

AKT
(visible)

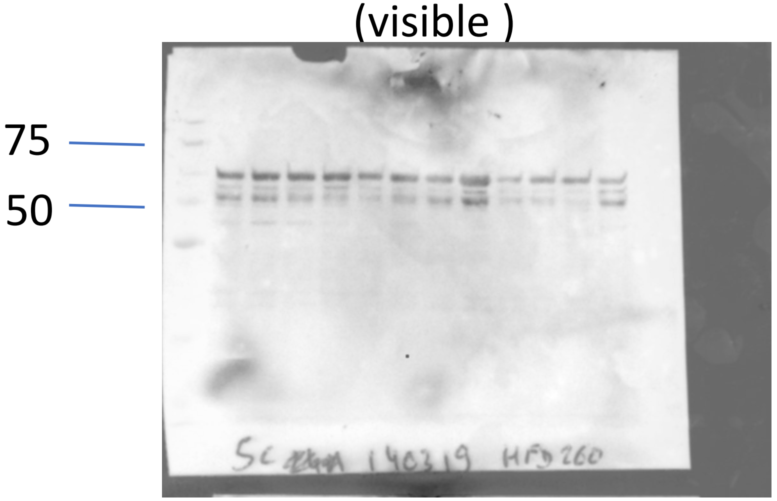

Figure S5d

pAKT

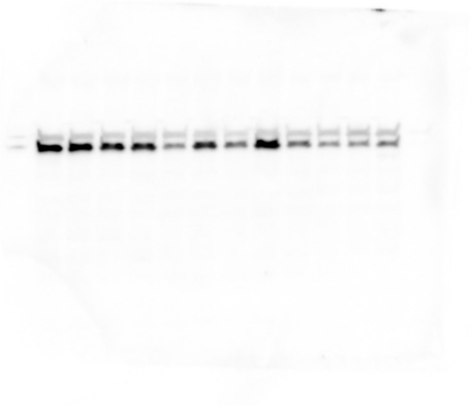

pAKT
(visible)

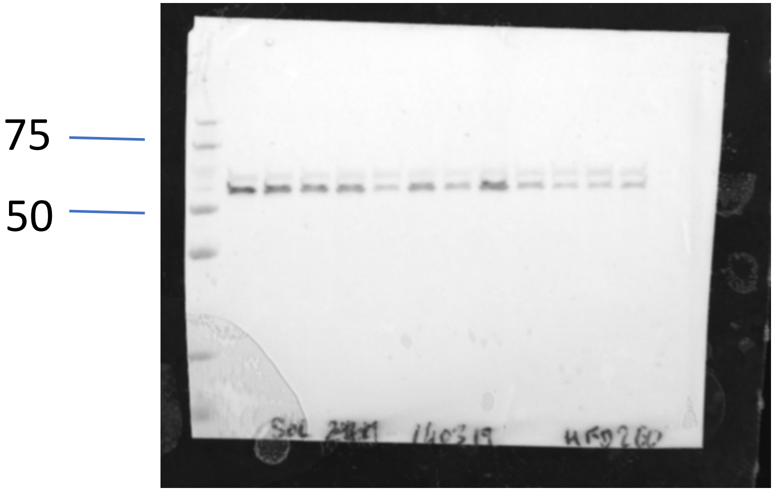

AKT

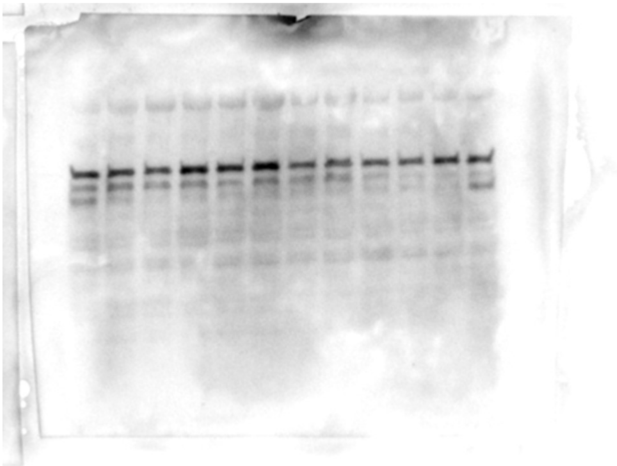

AKT
(visible)

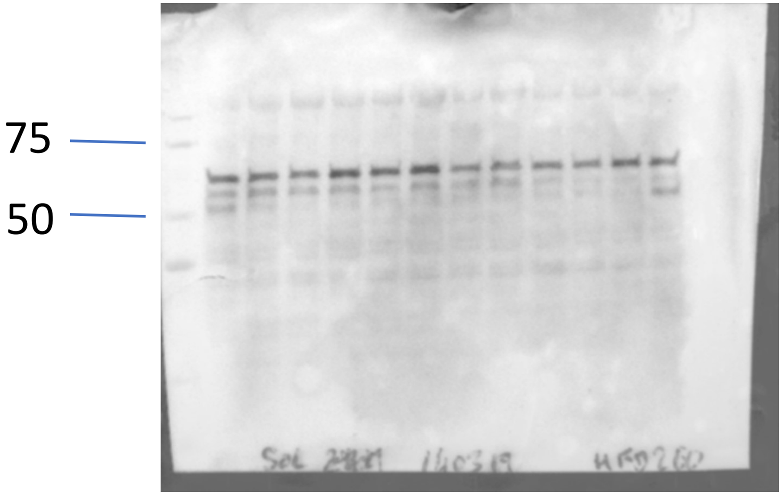

Figure S8a

pAKT

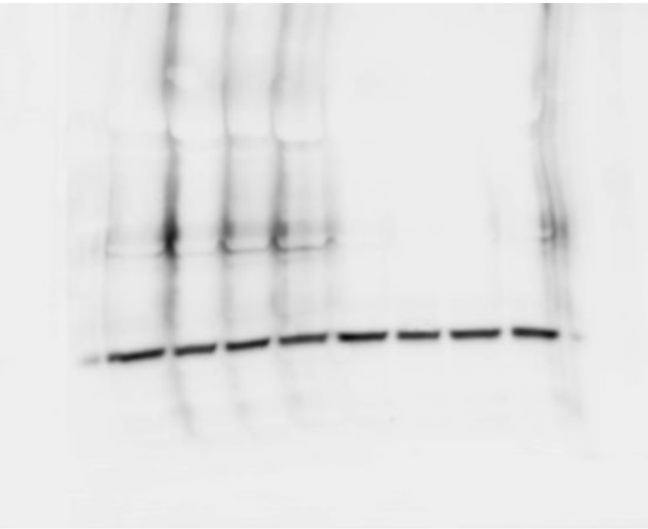

pAKT (visible)

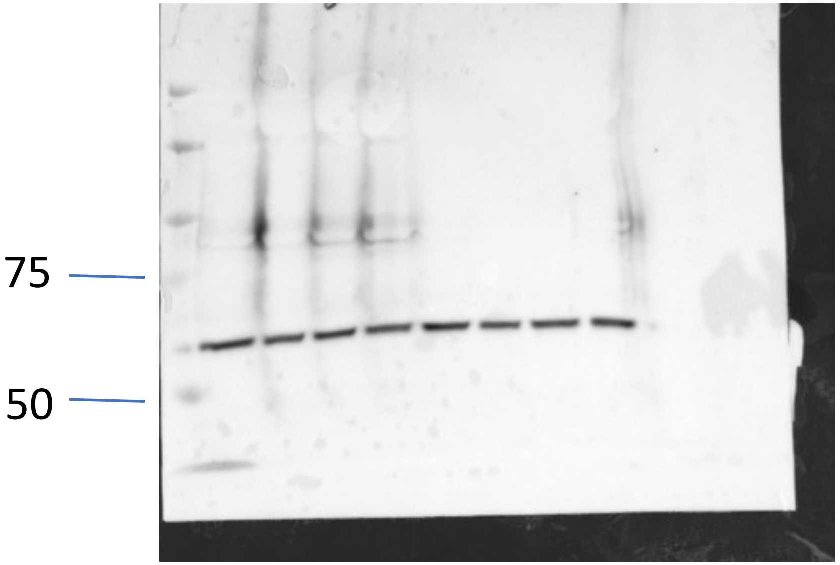

AKT

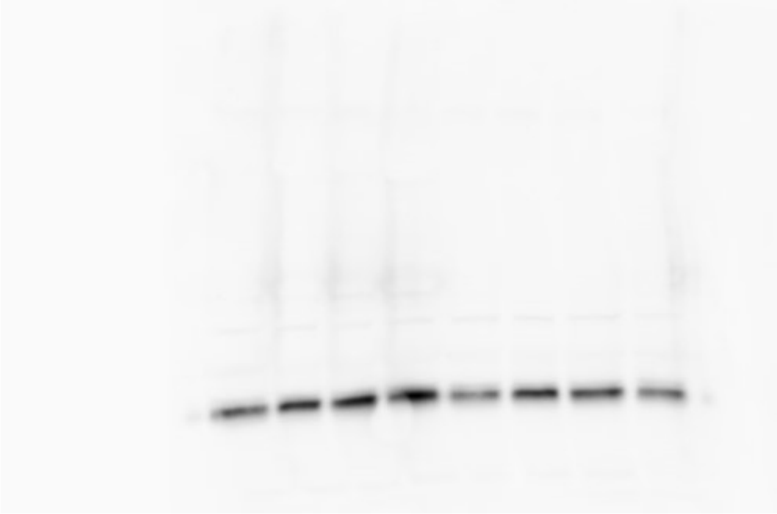

AKT (visible)

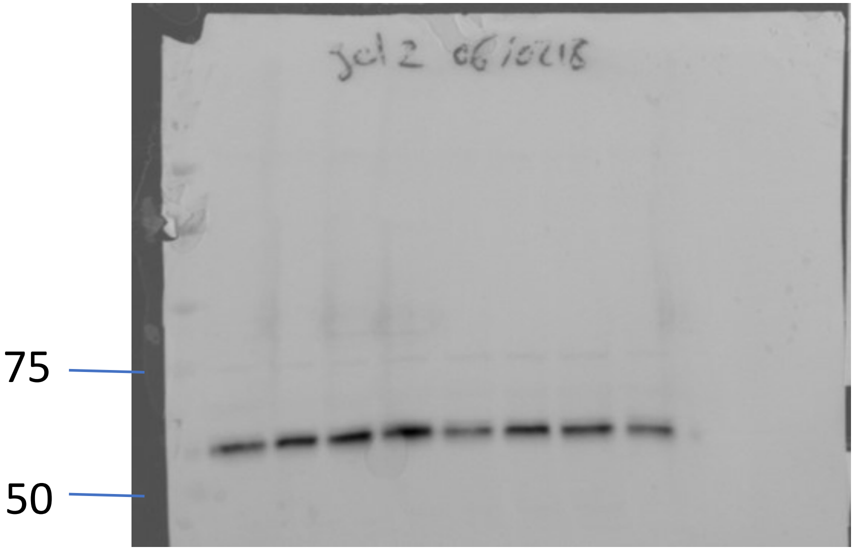

Figure S8b

pAKT

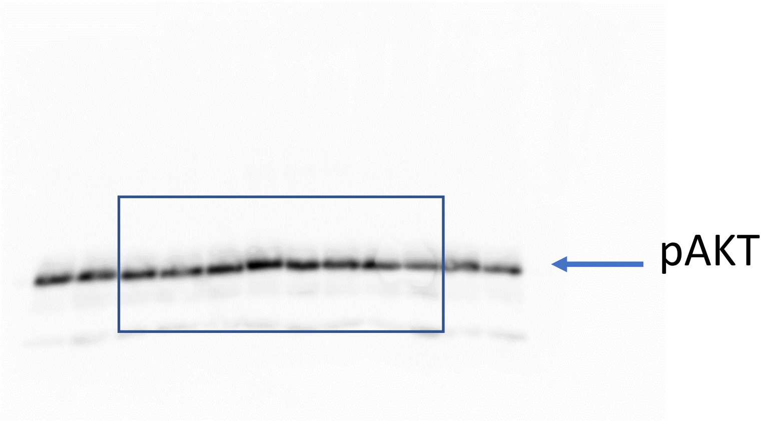

AKT

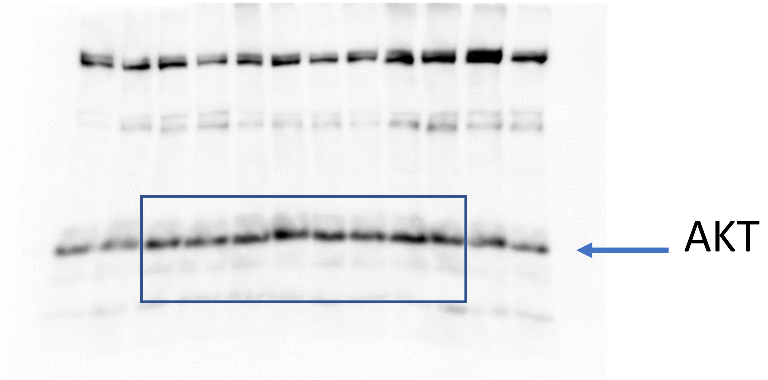

Figure S8b

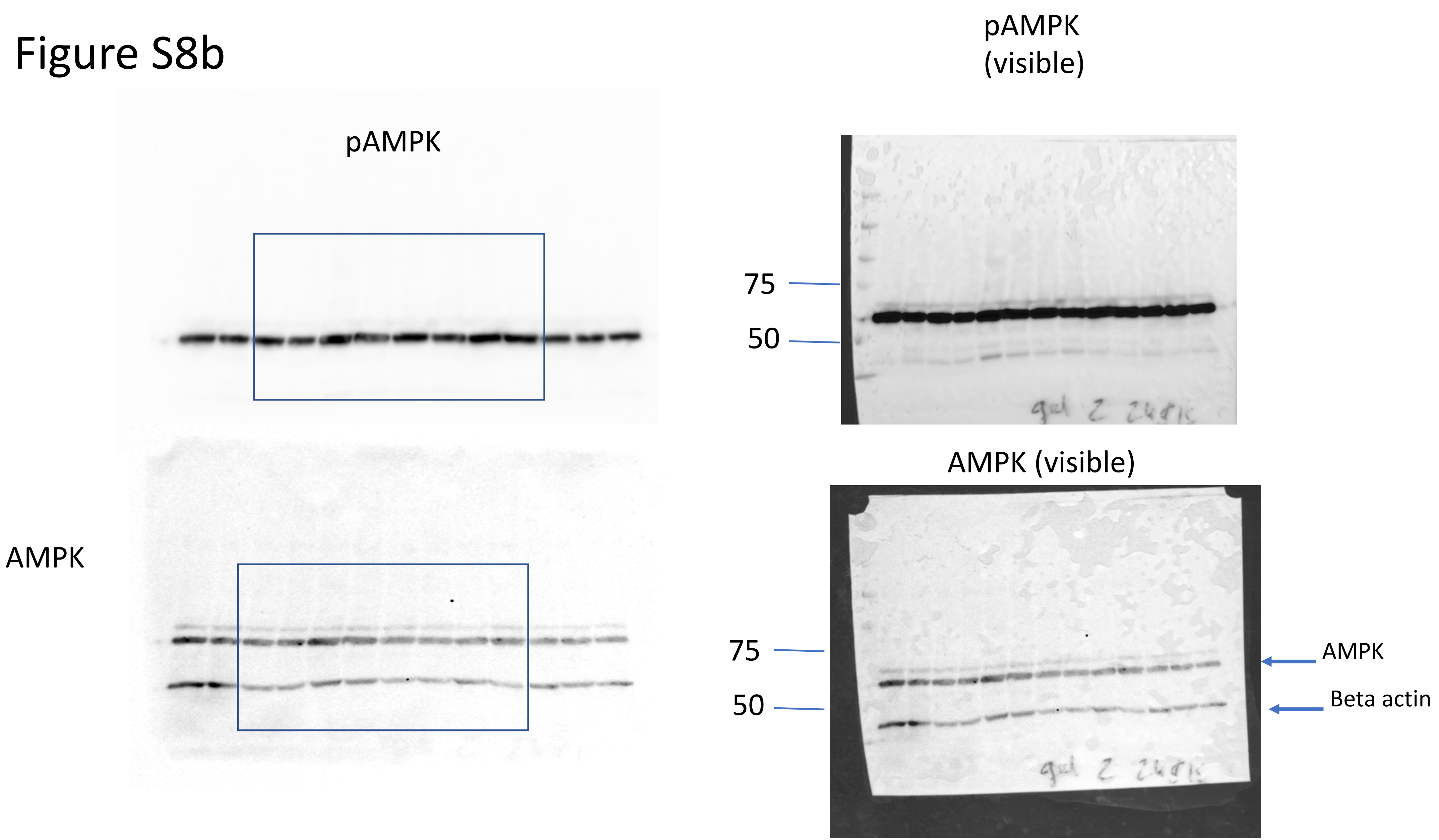
pAMPK
AMPK
pAMPK
(visible)
75
50
gel 2 24 8 K
AMPK (visible)
75
50
AMPK
Beta actin
gel 2 24 8 K

Figure S11a

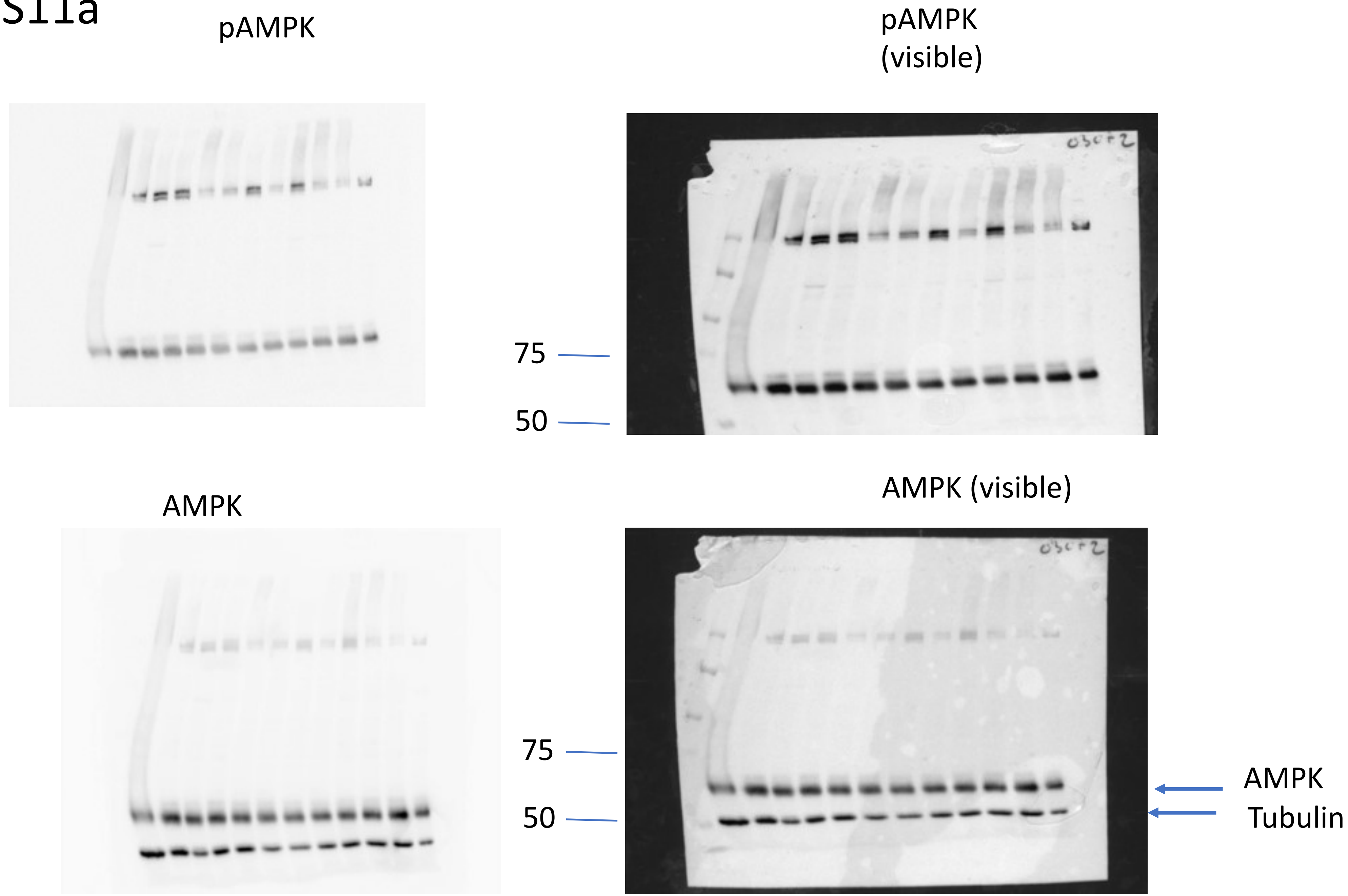

pAMPK
pAMPK
(visible)
75
50
AMPK
AMPK (visible)
75
50
AMPK
Tubulin

Figure S11 b

pAMPK

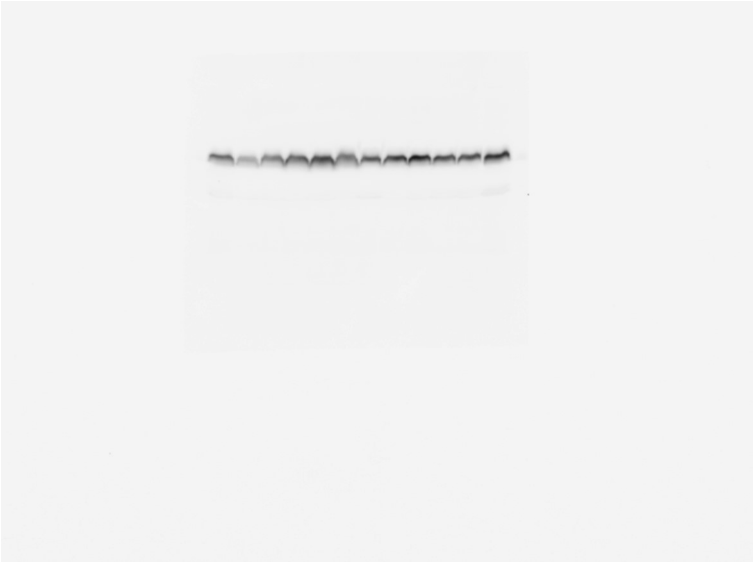

pAMPK
(visible)

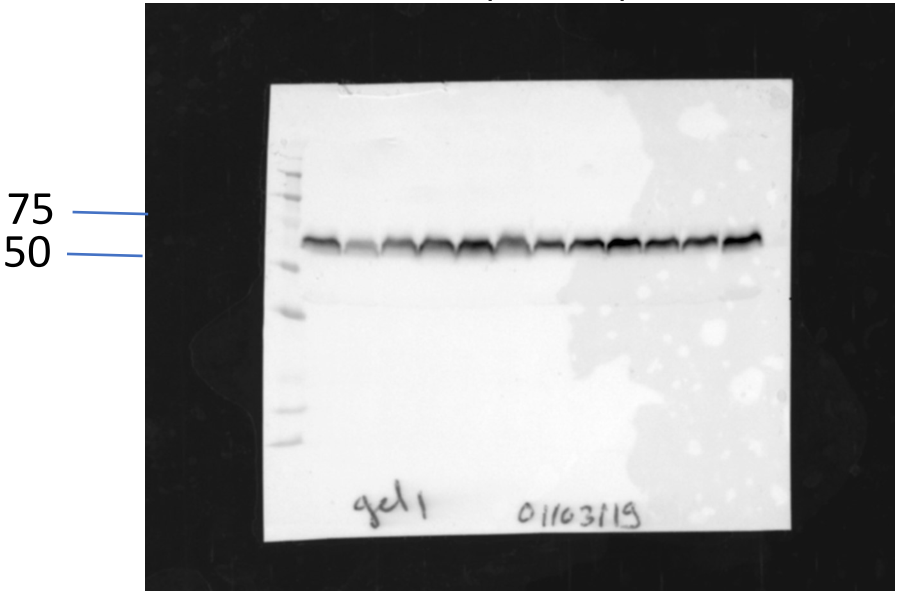

AMPK

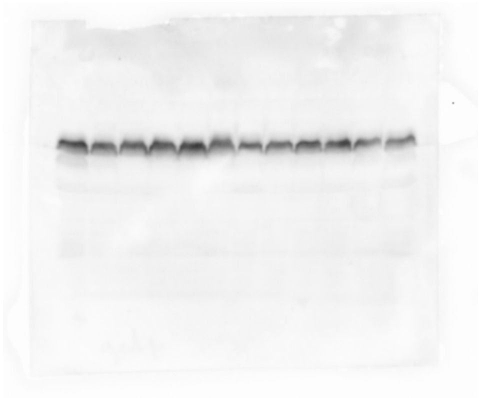

AMPK
(visible)

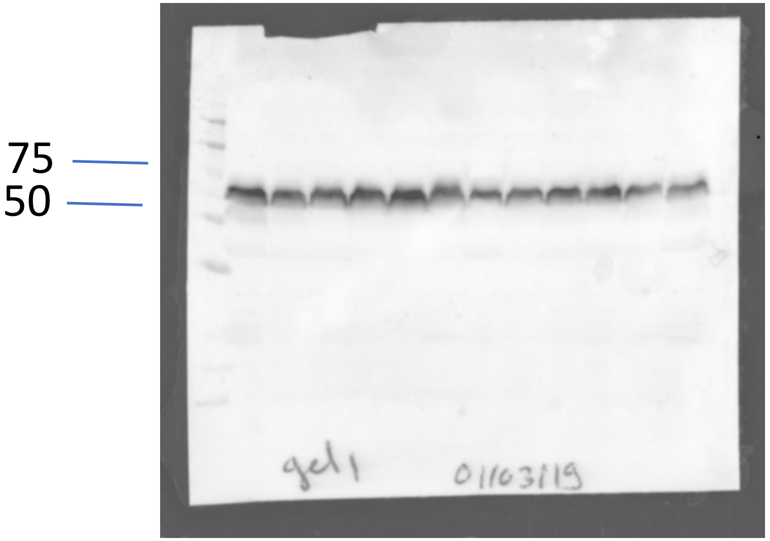

Figure S11 c

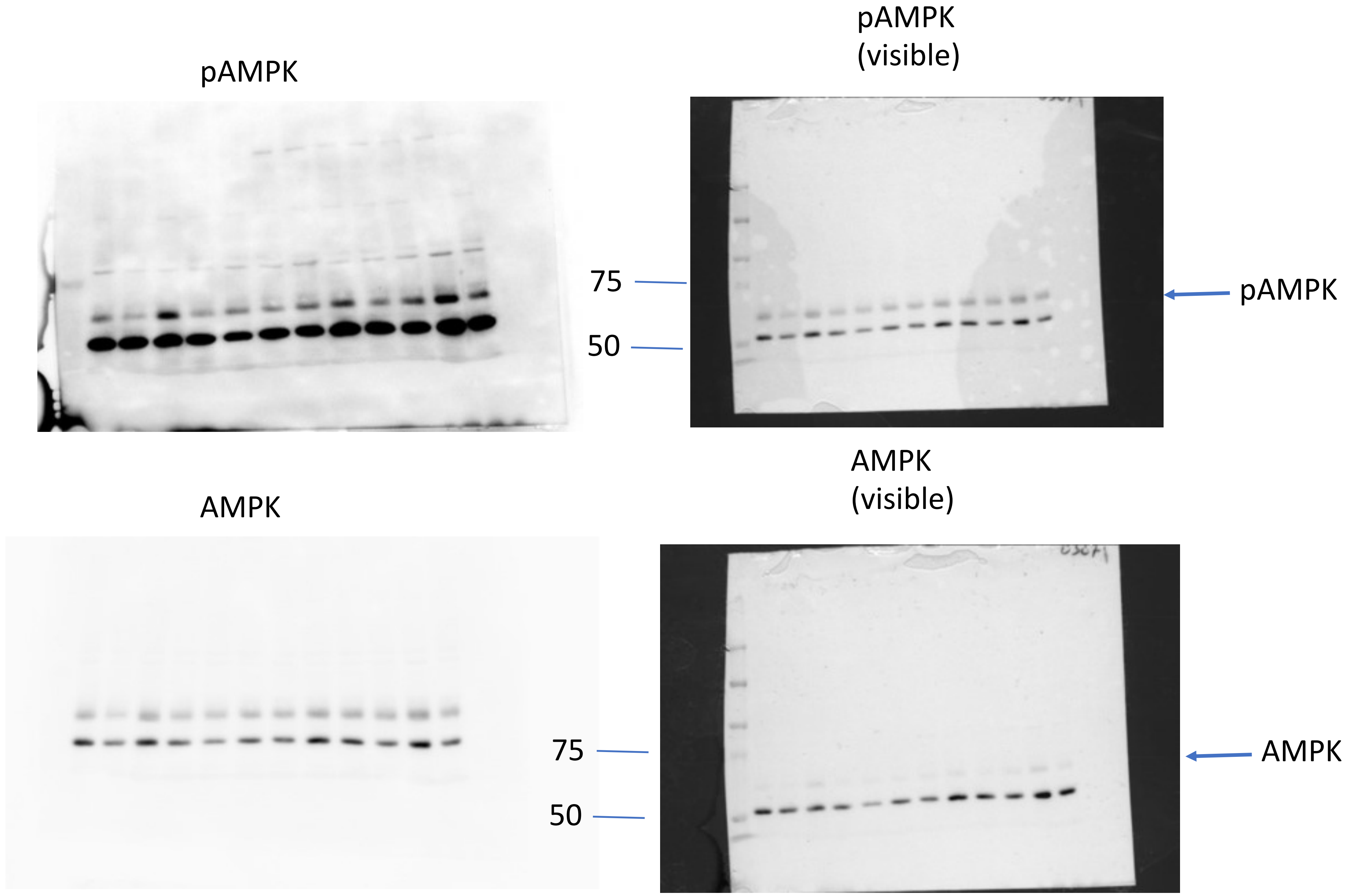

Figure S11d

pAMPK

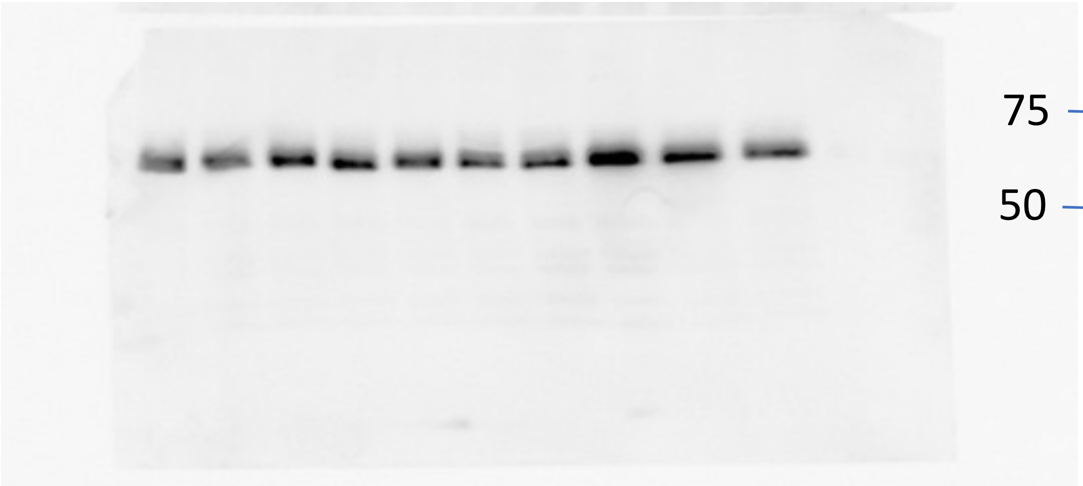

pAMPK
(visible)

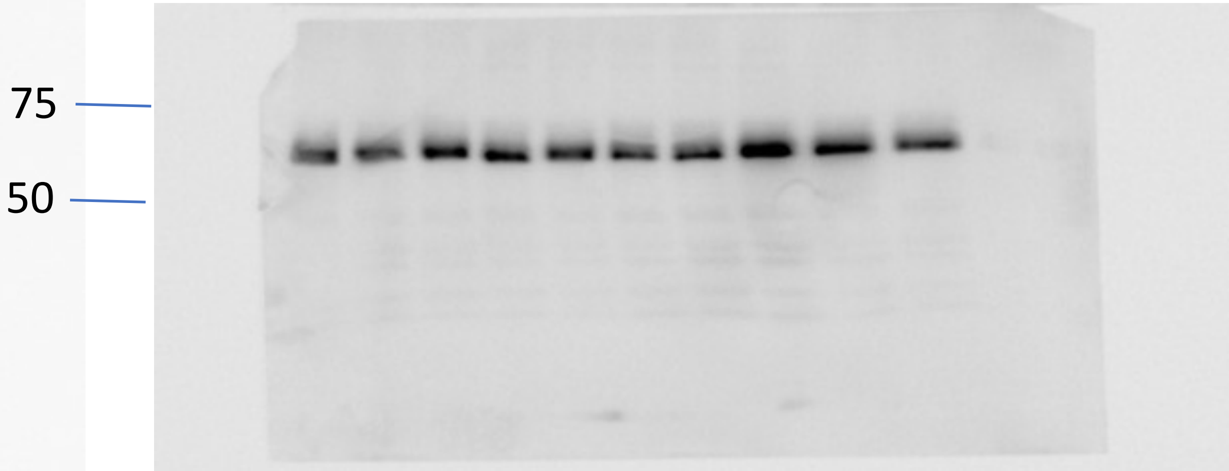

AMPK

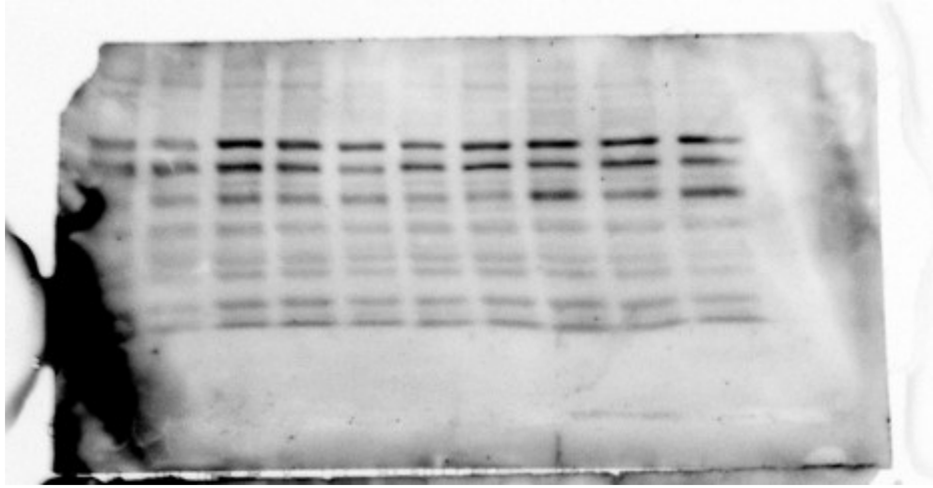

AMPK
(visible)

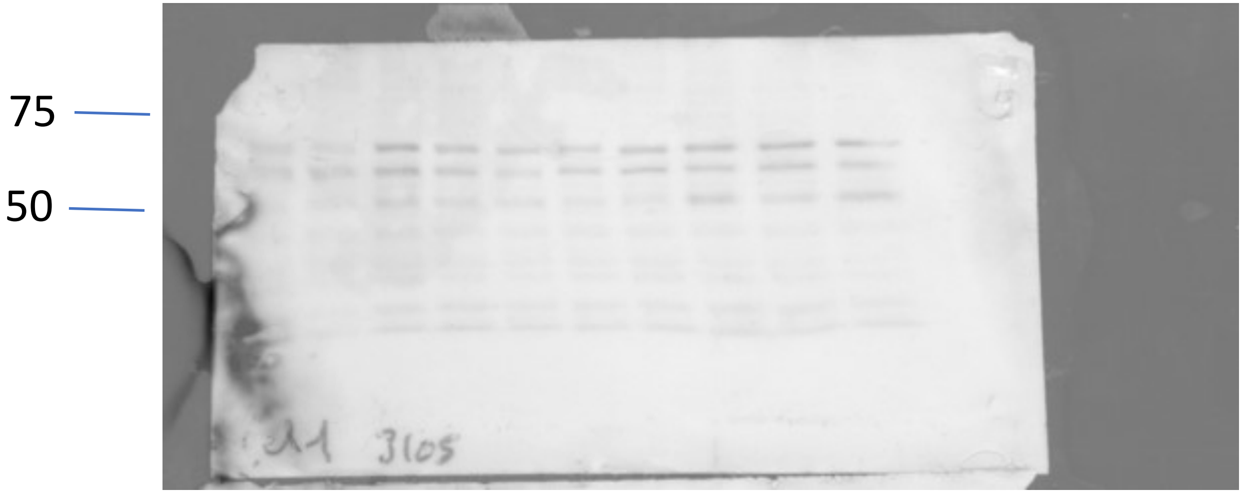

Supplement: Supplementary file 2 — Supplementary Information [file 42003_2023_4616_MOESM2_ESM.pdf]
