# Supplementary material for: Antimicrobial protein REG3A regulates glucose homeostasis and insulin resistance in obese diabetic mice
Source: Commun Biol. 2023 Mar 15;6:269. doi: 10.1038/s42003-023-04616-5 (PMC10015038; doi:10.1038/s42003-023-04616-5)
Supplement: Supplementary file 3 — Description of Additional Supplementary Files [file 42003_2023_4616_MOESM3_ESM.pdf]

Description of Additional Supplementary Files

File name: Supplementary Data 1
Description: Numerical source data of Figure 1

File name: Supplementary Data 2
Description: Numerical source data of Figure 2

File name: Supplementary Data 3
Description: Numerical source data of Figure 3

File name: Supplementary Data 4
Description: Numerical source data of Figure 4

File name: Supplementary Data 5
Description: Numerical source data of Figure 5

File name: Supplementary Data 6
Description: Numerical source data of Figure 6

File name: Supplementary Data 7
Description: Numerical source data of Figure 7
